# Supplementary material for: Qualitative Factor-Based Comparison of NMR, Targeted and Untargeted GC-MS and LC-MS on the Metabolomic Profiles of Rioja and Priorat Red Wines
Source: Foods. 2020 Sep 29;9(10):1381. doi: 10.3390/foods9101381 (PMC7600871; doi:10.3390/foods9101381)
Supplement: Supplementary file 1 [file foods-09-01381-s001.zip › supplementary_figures_tables 2/TableS6.pdf]

Table S6: GC-MS metabolites, with statistical and practical significance, for cellar ICVV. Numbers at the end of the acronyms represent sampling month. Values represent the area of the detected chromatographic peaks after applying variance stabilization. The metabolites can be grouped in the following chemical classes (1) benzene and substituted derivatives, (2) carbonyl compounds, (3) carboxylic acids and derivatives, (4) fatty acyls, (5) hydroxy acids and derivatives, (6) indoles and derivatives, (7) keto acids and derivatives, (8) lactones, (9) olefins, (10) oxolanes, (11) phenols, (12) phenylpropanoic acids, (13) phenol lipids, (14) saturated hydrocarbons, (15) stilbenes, (16) tetrahydrofurans, (17) thiolanes, (18) unsaturated hydrocarbons.

**Compound**	**FML_0**	**BAO_3**	**BAO_9**	**BAO_12**	**BAN_3**	**BAN_9**	**BAN_12**	**BTO_12**	**BTN_12**
2,4-di-tert-butylphenol[1D]	2,41E+06	3,08E+06	4,83E+05	1,64E+06	1,50E+06	4,56E+05	9,09E+05	5,62E+05	5,33E+05
2-hydroxy-3,4-dimethoxybenzoic acid[1U]	7,97E+05	1,24E+06	1,72E+06	7,66E+05	2,26E+06	2,49E+06	1,44E+06	5,91E+05	8,29E+05
2-phenethyl acetate[1D]	1,77E+06	1,07E+06	6,00E+05	3,33E+05	1,28E+06	5,62E+05	4,41E+05	3,63E+05	4,18E+05
4-nonylphenol[1U]	4,40E+05	4,30E+05	9,84E+05	5,85E+05	4,52E+05	9,89E+05	8,52E+05	5,39E+05	5,57E+05
benzyl alcohol[1U]	2,65E+05	1,49E+05	1,77E+06	9,84E+05	4,07E+05	1,59E+06	1,44E+06	1,10E+06	1,14E+06
dibutyl phthalate[1D]	1,44E+06	1,33E+06	2,23E+05	8,11E+05	1,26E+06	2,83E+05	4,18E+05	4,09E+05	5,38E+05
diphenylketone[1D]	1,22E+06	7,78E+05	2,54E+05	4,33E+05	7,68E+05	3,27E+05	3,01E+05	5,09E+05	6,44E+05
ethyl-4-hydroxybenzoate[1U]	1,79E+05	5,17E+05	7,79E+05	4,23E+05	2,67E+05	4,01E+05	3,70E+05	5,37E+05	3,98E+05
methyl,2-methoxy-2-phenylacetate[1D]	2,29E+05	9,65E+04	9,09E+04	1,17E+05	8,45E+04	1,91E+05	1,80E+05	2,57E+04	2,87E+04
2-isopropyl-5-methyl-2-hexenal[2D]	5,16E+05	5,38E+05	1,34E+05	3,17E+05	4,45E+05	1,43E+05	2,12E+05	3,73E+05	3,72E+05
ethyl 5-oxo-dl-prolinate[3U]	2,26E+06	4,94E+06	7,92E+06	5,94E+06	4,56E+06	6,39E+06	6,91E+06	5,25E+06	5,05E+06
hexadecyl trichloroacetate[3U]	2,87E+05	1,24E+05	2,55E+05	5,54E+05	1,88E+05	2,89E+05	2,74E+06	7,56E+05	7,89E+05
l,l-cyclo(leucylprolyl)[3U]	1,69E+05	2,66E+05	4,12E+05	2,41E+05	2,46E+05	4,33E+05	3,30E+05	2,78E+05	2,95E+05
methionol[3U]	3,07E+05	2,20E+06	4,68E+06	2,79E+06	7,48E+05	1,83E+06	1,58E+06	2,47E+06	3,84E+06
oxalic acid dibutyl ester[3U]	2,98E+05	1,70E+05	5,16E+05	5,78E+05	2,98E+05	7,47E+05	1,44E+06	1,19E+06	1,76E+06
1-dodecanol[4U]	4,29E+06	3,66E+05	4,84E+06	4,44E+06	3,25E+05	5,00E+06	4,35E+06	5,26E+06	4,58E+06
ethyl octanoate[4D]	4,34E+06	3,96E+06	4,47E+06	4,26E+06	3,58E+06	6,50E+05	4,31E+06	4,59E+06	4,33E+06
ethyl undecanoate[4D]	5,46E+05	4,44E+05	4,84E+05	3,48E+05	4,45E+05	4,63E+05	2,42E+05	3,35E+05	3,24E+05
palmitic acid[4U]	3,55E+06	3,97E+05	9,47E+06	3,07E+06	4,05E+06	4,08E+07	4,99E+06	4,78E+07	4,86E+07
stearic acid[4U]	9,74E+05	6,44E+04	5,27E+06	3,47E+06	6,31E+04	7,06E+06	2,38E+06	4,05E+07	4,09E+07
valeric acid[4D]	4,75E+06	3,83E+06	4,36E+06	9,93E+05	4,39E+06	2,06E+06	2,47E+06	7,72E+05	6,35E+05
diethyl malate[5U]	5,36E+05	5,35E+06	5,57E+06	5,55E+06	5,53E+06	5,09E+06	5,37E+06	7,76E+05	7,57E+05
ethyl-3-hydroxybutyrate[5D]	5,87E+06	3,26E+06	5,05E+06	5,68E+05	5,88E+06	9,96E+05	5,51E+06	5,56E+05	5,90E+05
d-tryptophan[6U]	9,49E+04	8,39E+04	2,58E+05	5,56E+05	5,21E+05	2,08E+05	2,04E+05	5,54E+05	5,47E+05
indole-3-methyl acetate[6D]	3,07E+05	2,21E+05	1,21E+05	1,34E+05	2,16E+05	1,31E+05	5,71E+04	1,09E+05	6,16E+04
tryptophol[6U]	2,30E+07	2,84E+07	1,04E+08	5,03E+07	4,10E+07	7,89E+07	8,59E+07	5,76E+07	5,74E+07
oxoglutaric acid[7U]	2,87E+05	2,33E+05	2,71E+05	1,42E+06	1,88E+05	2,17E+05	2,74E+06	7,17E+05	5,35E+05
5-ethoxyoxolan-2-one[8D]	8,96E+06	1,06E+05	2,52E+06	5,88E+05	3,39E+06	3,11E+06	1,08E+06	7,26E+04	6,52E+04
gamma-butyrolactone[8U]	1,44E+07	2,21E+07	3,81E+07	2,60E+07	2,11E+07	3,04E+07	3,49E+07	2,26E+07	2,15E+07
1-tetradecene[9D]	5,96E+05	9,18E+05	2,73E+05	3,71E+05	4,86E+05	3,49E+04	1,98E+05	8,38E+04	4,46E+04
succinic anhydride[10D]	2,05E+07	1,91E+06	3,48E+06	3,44E+06	7,02E+06	2,10E+06	9,74E+05	1,76E+06	1,05E+06
2-methoxy-4-vinylphenol[11D]	6,21E+05	5,57E+05	4,36E+05	2,26E+05	5,52E+05	2,76E+05	2,93E+05	2,83E+05	2,84E+05

homovanillic acid[11U]	1,17E+05	2,53E+05	4,91E+05	2,10E+05	2,08E+05	4,61E+05	3,56E+05	1,88E+05	1,92E+05
tyrosol[11U]	7,44E+06	9,68E+06	3,49E+07	2,63E+07	1,44E+07	3,24E+07	1,99E+07	2,36E+07	2,29E+07
methyl,3-(4-hydroxyphenyl) propanoate[12U]	3,74E+05	7,97E+05	1,67E+06	1,00E+06	8,38E+05	1,59E+06	1,60E+06	9,25E+05	8,23E+05
phenol, 2-(2h-benzotriazol-2-yl)-4,6-bis (1,1-dimithylpropyl)[12D]	1,292E+06	9,90E+05	1,47E+06	7,85E+05	1,32E+06	1,55E+06	7,18E+05	3,66E+05	6,47E+05
borneol[13D]	5,85E+05	4,72E+05	4,56E+05	2,47E+05	4,96E+05	5,48E+05	4,35E+05	2,74E+05	2,77E+05
carvacrol[13D]	4,01E+05	3,72E+05	1,33E+05	1,95E+05	2,15E+05	1,45E+05	1,87E+05	1,71E+05	2,25E+05
2,10-dimethylundecane[14U]	8,45E+04	1,64E+05	7,69E+05	3,97E+05	4,74E+04	4,75E+05	3,99E+05	5,80E+04	5,65E+04
2,7,10-trimethyldodecane[14D]	1,77E+06	9,45E+05	2,14E+05	7,17E+05	1,40E+06	6,14E+05	7,81E+05	1,50E+06	1,20E+06
3,8-dimethylundecane[14U]	2,22E+05	2,89E+05	1,11E+05	3,50E+05	2,13E+05	1,02E+05	1,81E+05	3,46E+05	3,18E+05
isoheptane[14D]	2,47E+06	2,10E+06	8,16E+05	1,69E+06	1,86E+06	6,71E+05	1,14E+06	2,19E+06	1,87E+06
1,2-diphenylcyclobutane[15D]	3,34E+05	3,46E+05	2,73E+05	1,78E+05	1,90E+05	3,18E+05	1,03E+05	1,23E+05	8,95E+04
7,9-di-tert-butyl-1-oxaspiro[4,5]deca-6,9-diene-2,8-dione[16D]	2,02E+06	1,91E+06	5,92E+05	7,06E+05	1,54E+06	6,20E+05	1,01E+06	4,00E+05	3,85E+05
2-methyltetrahydrothiophen-3-one[17U]	3,98E+06	3,89E+06	3,69E+07	1,73E+07	5,17E+06	2,73E+07	2,30E+07	1,78E+07	1,78E+07
4,6,8-trimethylnon-1-ene[18D]	1,06E+06	7,24E+05	5,81E+05	7,27E+05	9,73E+05	4,53E+05	7,95E+05	1,48E+06	1,66E+06
5-eicosene[18U]	2,74E+05	1,41E+05	3,08E+05	5,90E+05	1,74E+05	3,22E+05	4,30E+05	7,64E+05	7,92E+05
6-tridecene[18U]	1,85E+05	5,70E+05	2,05E+06	2,60E+05	1,01E+05	2,13E+06	7,00E+05	8,03E+06	7,02E+06
