# Supplementary material for: Qualitative Factor-Based Comparison of NMR, Targeted and Untargeted GC-MS and LC-MS on the Metabolomic Profiles of Rioja and Priorat Red Wines
Source: Foods. 2020 Sep 29;9(10):1381. doi: 10.3390/foods9101381 (PMC7600871; doi:10.3390/foods9101381)
Supplement: Supplementary file 1 [file foods-09-01381-s001.zip › supplementary_figures_tables 2/TableS5.pdf]

Table S5: GC-MS metabolites, with statistical and practical significance, for cellar FB. Numbers at the end of the acronyms represent sampling month. Values represent the area of the detected chromatographic peaks after applying variance stabilization. The metabolites can be grouped in the following chemical classes (1) amines, (2) benzene and substituted derivatives, (3) carboxylic acids, (4) fatty acyls, (5) hydroxy acids, (6) indoles, (7) keto acids, (8) lactones, (9) phenols, (10) saturated hydrocarbons, (11) unsaturated hydrocarbons. Metabolites that exhibited increasing tendency are indicated as (U) and those with decreasing tendency as (D), whereas metabolites with relatively stable concentrations but with a peak on the 6th month are denoted as (S).

Compound	BAN_0	BAN_3	BAN_6	BAN_9	BAN_12	BAO_0	BAO_3	BAO_6	BAO_9
n-phenethylacetamide[1S]	2,85E+05	1,94E+05	5,38E+05	8,35E+04	2,83E+05	1,51E+05	1,34E+05	5,77E+05	8,38E+04
2,4-di-tert-butylphenol[2S]	3,72E+06	2,70E+06	6,25E+06	2,40E+06	1,41E+06	3,41E+06	3,44E+06	6,98E+06	2,55E+06
2-phenethyl acetate[2D]	8,86E+06	7,81E+06	3,74E+06	5,75E+06	2,98E+06	9,37E+06	8,37E+06	4,48E+06	5,08E+06
3,5-bis(1,1-dimethylethyl)- -4-hydroxy- benzoic acid ethyl ester[2S]	1,62E+05	8,74E+04	5,09E+05	5,44E+04	2,83E+05	8,55E+04	1,31E+05	4,92E+05	6,94E+04
ethyl,5-oxo-dl-prolinate[3U]	4,02E+06	9,37E+06	6,61E+06	1,64E+07	8,97E+06	4,77E+06	8,51E+06	7,60E+06	1,42E+07
2-butyl-1-octanol[4S]	7,99E+05	3,64E+05	1,81E+06	1,24E+05	4,39E+06	6,23E+05	5,33E+05	2,05E+06	2,83E+05
diethyl succinate[4U]	3,20E+07	1,13E+08	9,06E+07	2,61E+08	2,17E+08	4,49E+07	1,36E+08	1,24E+08	2,45E+08
diethyl malate[5U]	1,84E+06	4,60E+06	3,52E+06	9,50E+06	5,62E+06	2,22E+06	4,97E+06	4,13E+06	8,46E+06
indole-3-methyl acetate[6D]	7,43E+05	6,89E+05	3,44E+05	5,19E+05	2,81E+05	9,43E+05	7,03E+05	4,25E+05	4,66E+05
oxoglutaric acid[7U]	3,76E+06	6,09E+06	5,39E+06	1,44E+07	1,18E+07	5,55E+06	5,32E+06	6,23E+06	1,16E+07
gamma-butyrolactone[8U]	3,68E+07	7,24E+07	5,10E+07	1,38E+08	1,02E+08	3,50E+07	7,65E+07	6,01E+07	1,18E+08
2,3-dimethylphenol[9U]	2,73E+07	2,24E+07	1,08E+07	2,43E+07	2,73E+07	2,93E+07	2,44E+07	1,30E+07	2,11E+07
acetaminophen[9D]	7,49E+05	1,09E+06	7,08E+05	1,49E+06	9,04E+04	7,20E+05	8,89E+05	6,91E+05	1,14E+06
2-methylnonane[10S]	3,79E+06	1,68E+06	6,90E+06	1,19E+06	4,38E+06	1,92E+06	1,51E+06	7,67E+06	1,46E+06
3-eicosyne[11S]	7,32E+05	3,70E+05	1,90E+06	2,73E+05	6,10E+05	3,80E+05	5,74E+05	2,50E+06	2,91E+05
4,6,8-trimethylnon-1-ene[11S]	2,06E+06	9,26E+05	4,64E+06	5,64E+05	3,01E+06	1,53E+06	1,51E+06	5,02E+06	6,46E+05
