# Supplementary material for: Qualitative Factor-Based Comparison of NMR, Targeted and Untargeted GC-MS and LC-MS on the Metabolomic Profiles of Rioja and Priorat Red Wines
Source: Foods. 2020 Sep 29;9(10):1381. doi: 10.3390/foods9101381 (PMC7600871; doi:10.3390/foods9101381)
Supplement: Supplementary file 1 [file foods-09-01381-s001.zip › supplementary_figures_tables 2/TableS4.pdf]

Table 3: LC-MS metabolites, with statistical and practical significance, for cellar ICVV. Numbers at the end of the acronyms represent sampling month. Values represent the area of the detected chromatographic peaks after applying variance stabilization. The metabolites can be grouped in the following chemical classes (1) benzene and substituted derivatives, (2) cinnamic acids and derivatives, (3) coumarins and derivatives, (4) flavonoids, (5) phenol ethers and (6) phenols. Metabolites that exhibited increasing tendency are indicated as (U) and those with decreasing tendency as (D).

Compound	FML_0	BAN_3	BAN_9	BAN_12	BAO_3	BAO_9	BAO_12	BTN_12
3,4-dihydroxybenzaldehyde[1U]	1,42E+05	3,85E+05	6,21E+05	6,20E+05	2,21E+05	3,81E+05	3,69E+05	4,50E+05
benzoic acid[1U]	1,34E+05	3,55E+05	4,02E+05	4,20E+05	2,94E+05	3,05E+05	3,08E+05	2,73E+05
ethyl gallate[1U]	9,75E+04	1,94E+05	4,17E+05	4,53E+05	2,11E+05	3,93E+05	4,43E+05	4,53E+05
gallic acid[1D]	5,90E+05	4,85E+05	1,03E+05	1,90E+05	4,91E+05	1,91E+05	1,90E+05	3,63E+05
vanillic acid[1D]	7,07E+04	3,28E+04	2,83E+04	3,19E+04	3,94E+04	2,81E+04	3,10E+04	3,26E+04
caffeic acid[2U]	4,49E+04	3,07E+05	4,64E+05	4,42E+05	2,75E+05	5,15E+05	5,36E+05	5,40E+05
scopoletin[3U]	7,79E+04	2,29E+05	4,74E+05	4,42E+05	1,82E+05	3,30E+05	3,21E+05	1,76E+05
6-methoxyluteolin[4U]	1,01E+05	5,64E+04	3,07E+05	2,07E+05	6,79E+04	1,45E+05	1,49E+05	9,61E+04
astilbin[4D]	2,39E+05	2,10E+05	2,40E+04	1,66E+04	2,20E+05	2,37E+04	1,25E+04	1,28E+04
dihydromyricetin 3-rhamnoside[4D]	5,12E+04	3,24E+04	2,72E+04	2,33E+04	2,81E+04	2,40E+04	1,77E+04	2,49E+04
dihydroquercetin[4D]	5,21E+05	2,73E+05	2,38E+05	1,75E+05	5,21E+05	2,06E+05	2,20E+05	1,98E+05
hyperoside[4D]	6,57E+06	4,65E+06	3,09E+06	2,21E+06	4,43E+06	2,81E+06	2,27E+06	2,10E+06
luteolin 7-glucoside[4D]	9,87E+05	4,83E+05	2,12E+05	1,41E+05	4,53E+05	1,90E+05	1,42E+05	1,33E+05
naringin[4D]	3,98E+05	1,78E+05	1,40E+05	1,21E+05	1,88E+05	1,51E+05	1,34E+05	1,27E+05
procyanidin C2[4D]	2,27E+05	1,36E+05	4,48E+04	3,38E+04	1,69E+05	7,35E+04	5,86E+04	3,87E+04
4-methoxyphenylacetic acid[5U]	5,71E+04	7,19E+04	1,14E+05	1,39E+05	1,18E+05	1,78E+05	1,80E+05	1,59E+05
syringaldehyde[6U]	5,74E+05	9,71E+05	1,51E+06	1,57E+06	1,40E+06	2,27E+06	2,25E+06	1,78E+06
