# Supplementary material for: Qualitative Factor-Based Comparison of NMR, Targeted and Untargeted GC-MS and LC-MS on the Metabolomic Profiles of Rioja and Priorat Red Wines
Source: Foods. 2020 Sep 29;9(10):1381. doi: 10.3390/foods9101381 (PMC7600871; doi:10.3390/foods9101381)
Supplement: Supplementary file 1 [file foods-09-01381-s001.zip › supplementary_figures_tables 2/supplementary_figures.pdf]

1. Supplementary figures

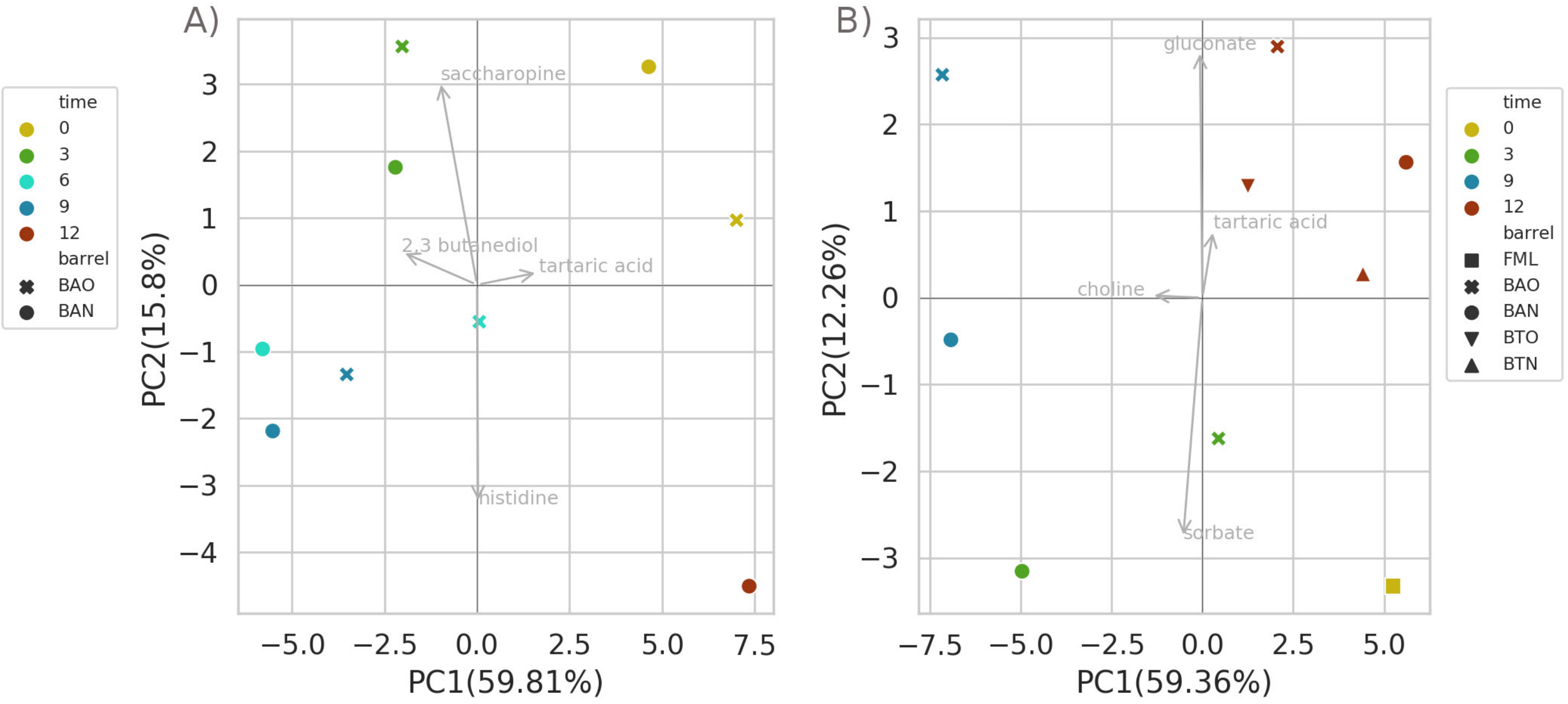

Figure S1: PCoA for cellars FB (A) and ICVV (B) based on the NMR metabolites. Metabolites with highest loadings or practical significance are shown. Acronyms BAN and BAO refer to new and old barrel respectively, FML to final malolactic fermentation stage and BTN and BTO to bottle-aged wine from new and old barrel respectively.

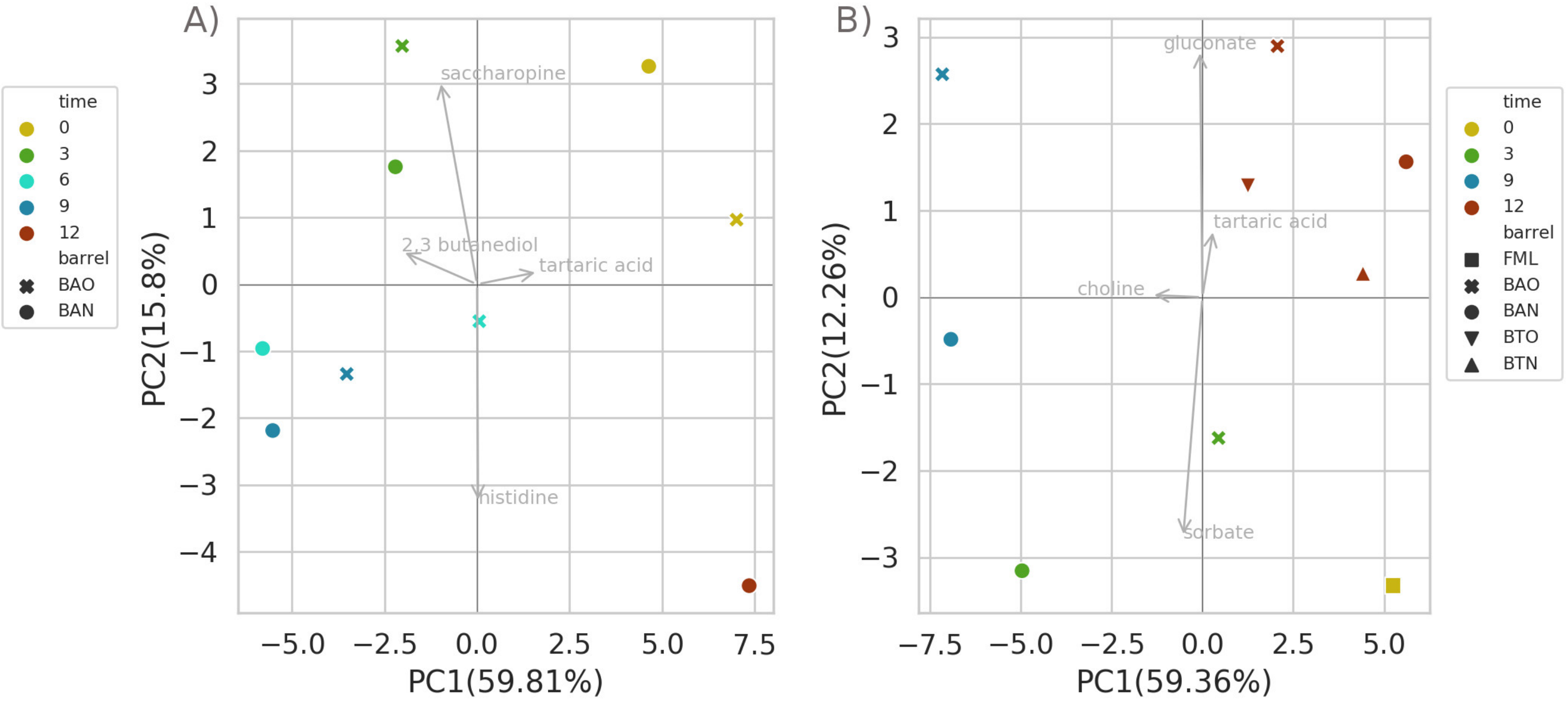

Figure S2: Log2 values of NMR metabolites with practical significance for the cellars FB (A-B) and ICVV (C-H). Acronyms BAN and BAO refer to barrel-aged wine from new and old barrel respectively, FML to final malolactic fermentation stage and BTN and BTO to bottle-aged wine from new and old barrel respectively.

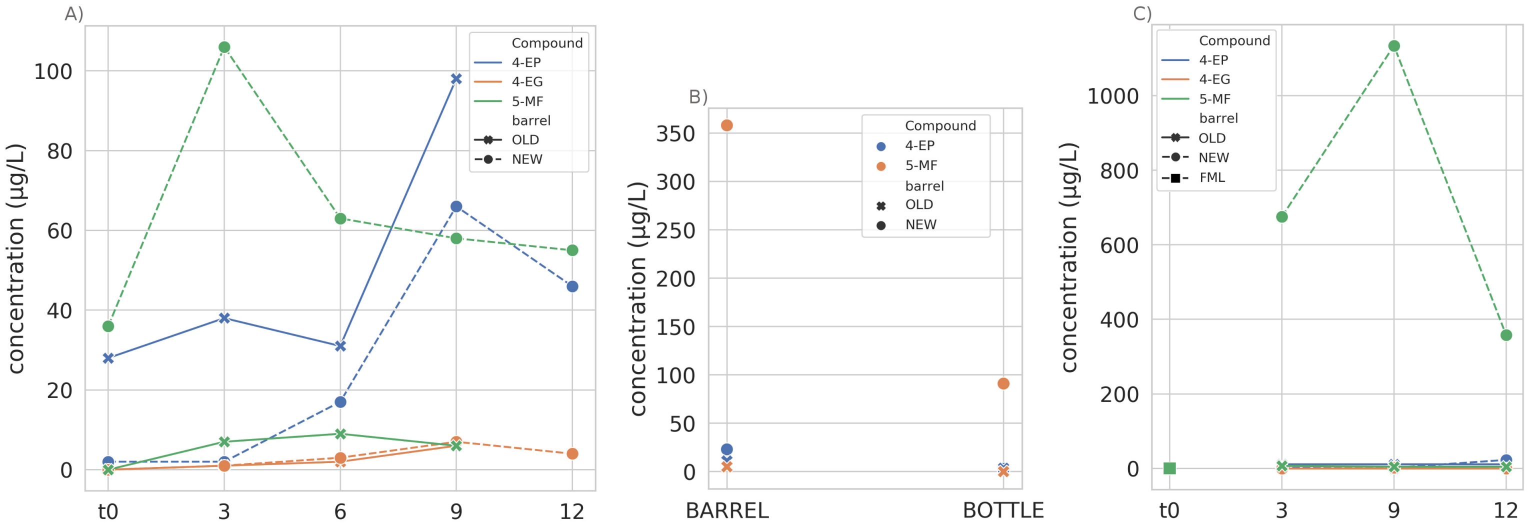

Figure S3: GC-MS targeted for the metabolites 4-ethylphenol (4-EP), 4-ethylguaiacol (4-EG) and 5-methylfurfural (5-MF) based on the factors barrel-type and time for cellar FB (A) and ICVV (C). Figure B concerns the comparison between the 12th month bottle and barrel-aged wine from ICVV cellar. For these samples the concentration of the 4-ethylguaiacol (4-EG) metabolite was zero.

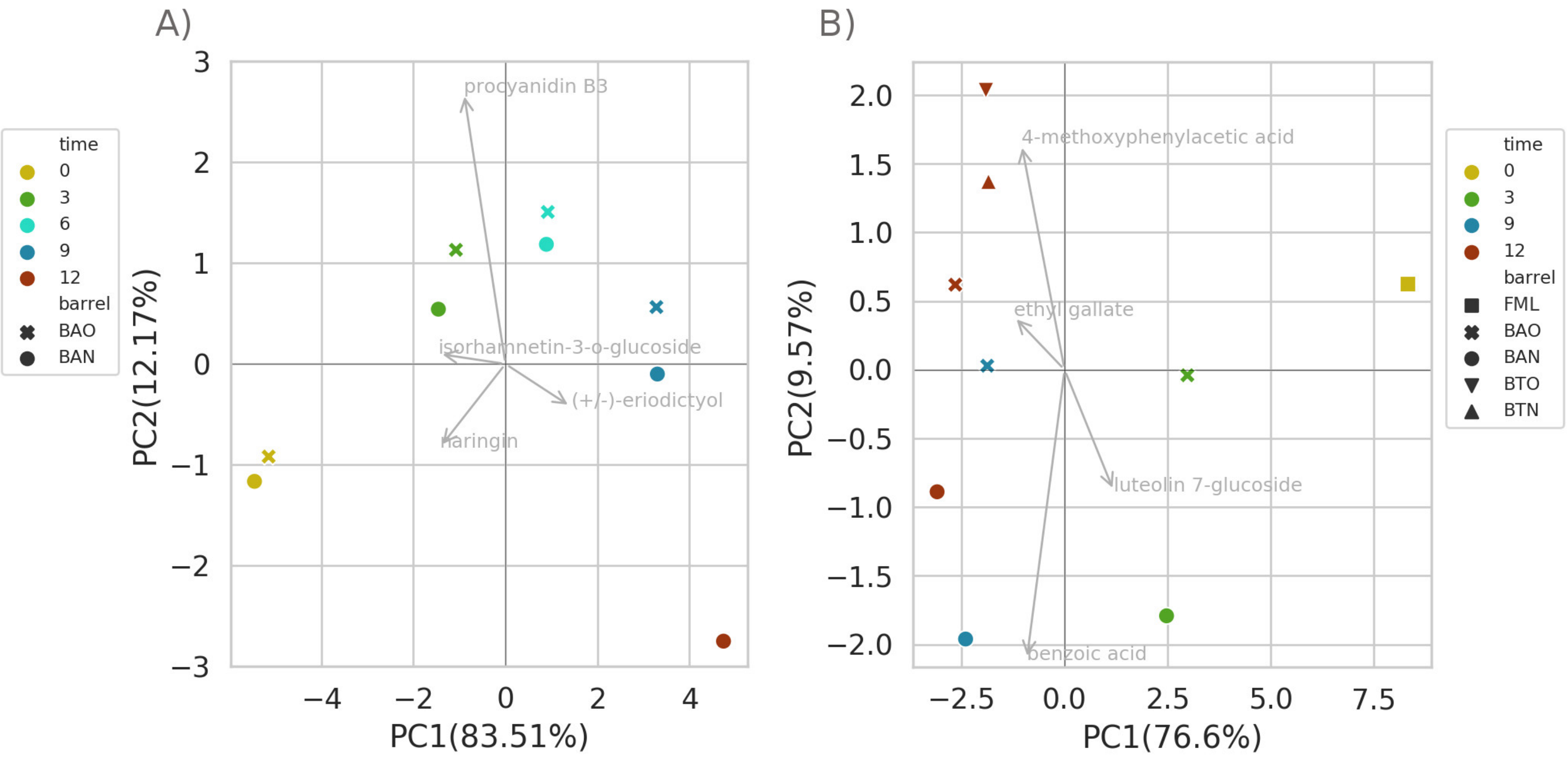

Figure S4: PCoA for cellars FB (A) and ICVV (B) based on the LC-MS metabolites. Metabolites with highest loadings acrosss the principal components are shown. Acronyms BAN and BAO refer to new and old barrel respectively, FML to final malolactic fermentation stage and BTN and BTO to bottle-aged wine from new and old barrel respectively.

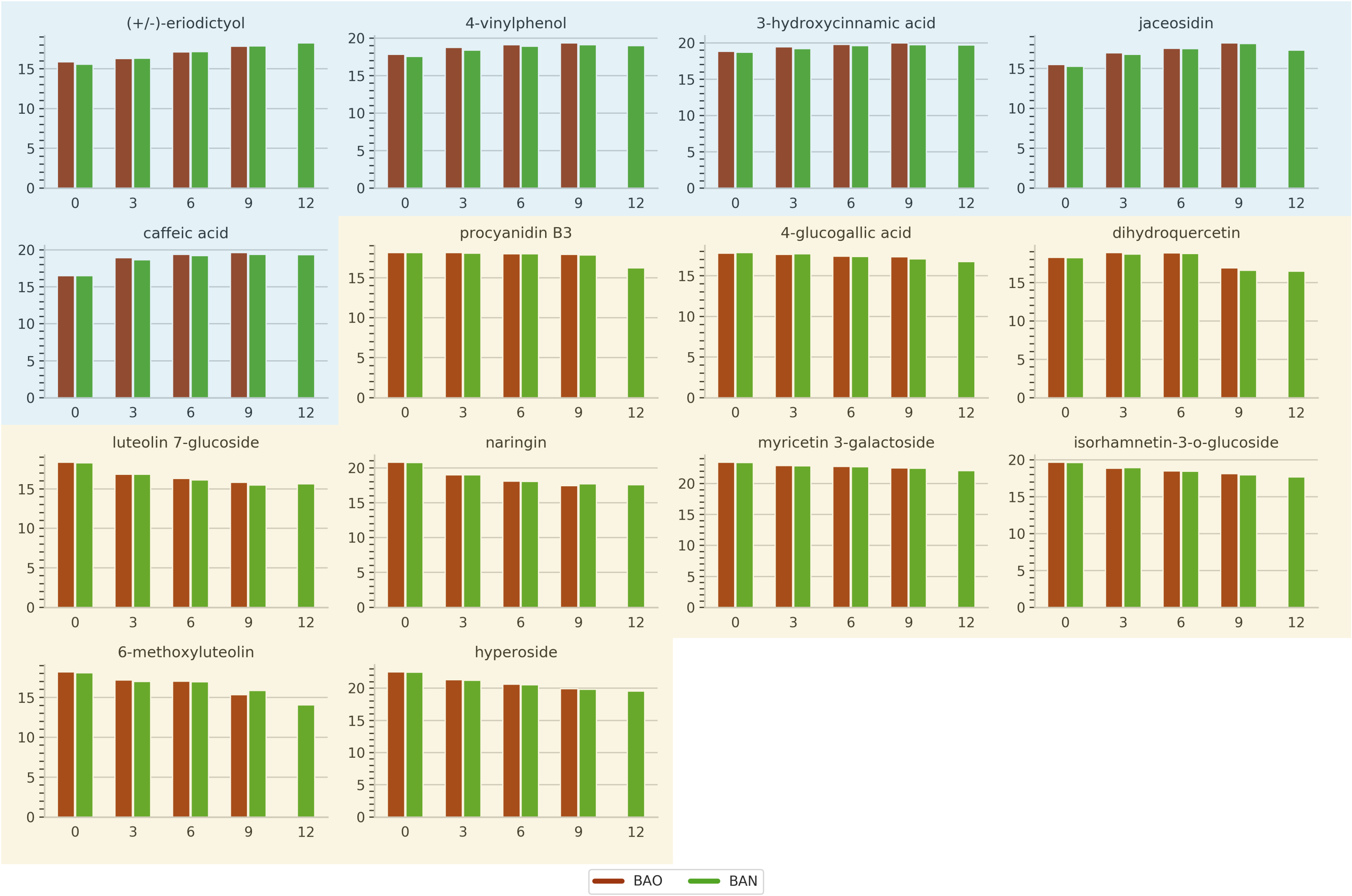

Figure S5: LC-MS log2 values of metabolites for cellar FB. Acronyms BAO and BAN refer to barrel-aged wine from old and new barrel respectively. Barplots of metabolites with blue background represent the group of metabolites whose concentration has an increasing trend whereas in orange background the group of metabolites with decreasing tendency.

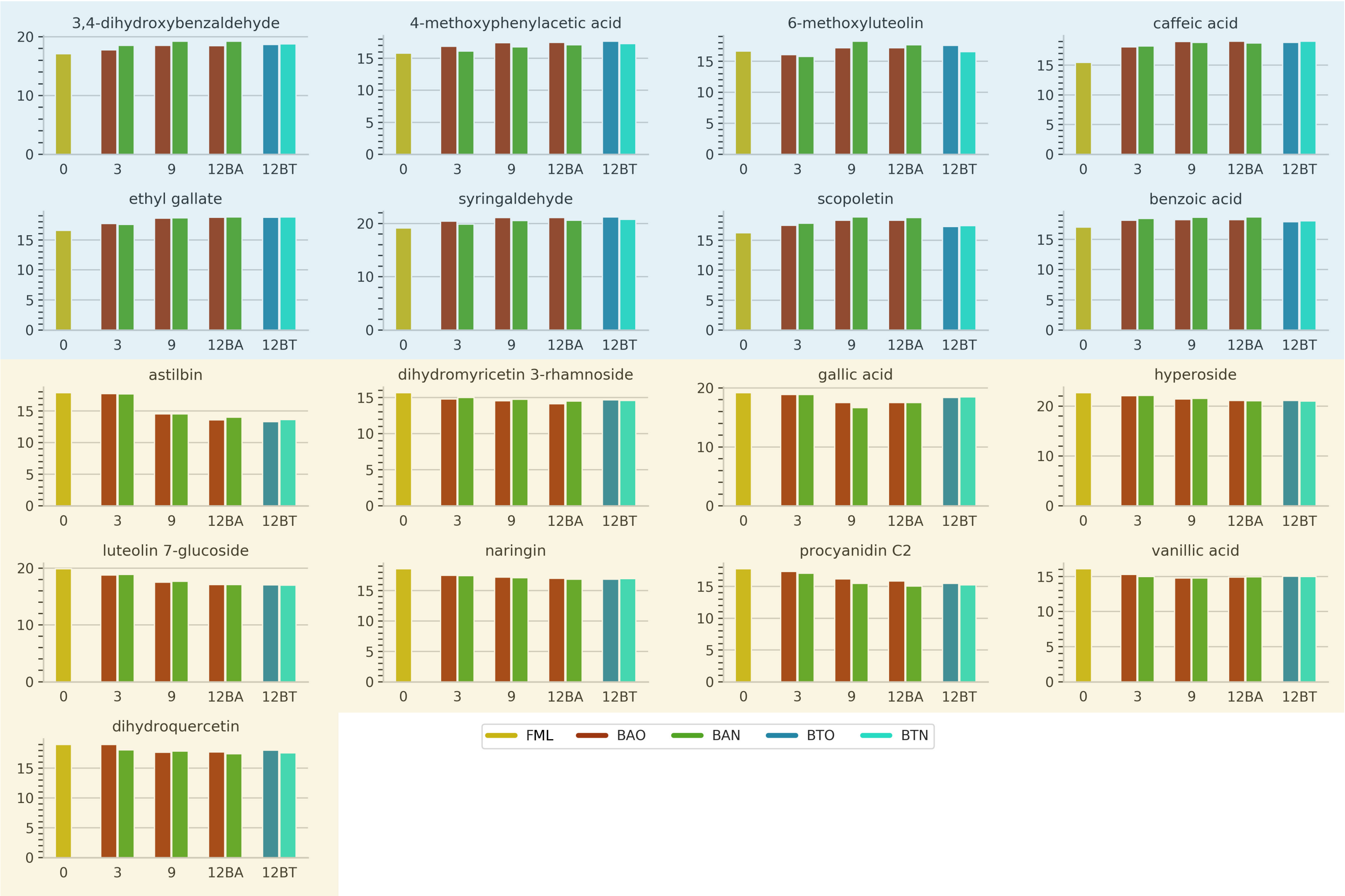

Figure S6: LC-MS log2 values of metabolites for cellar ICVV. Acronyms BAN and BAO refer to new and old barrel respectively, FML to final malolactic fermentation stage and BTN and BTO to bottle-aged wine from new and old barrel respectively. Barplots of metabolites with blue background represent the group of metabolites whose concentration has an increasing trend whereas in orange background the group of metabolites with decreasing tendency.

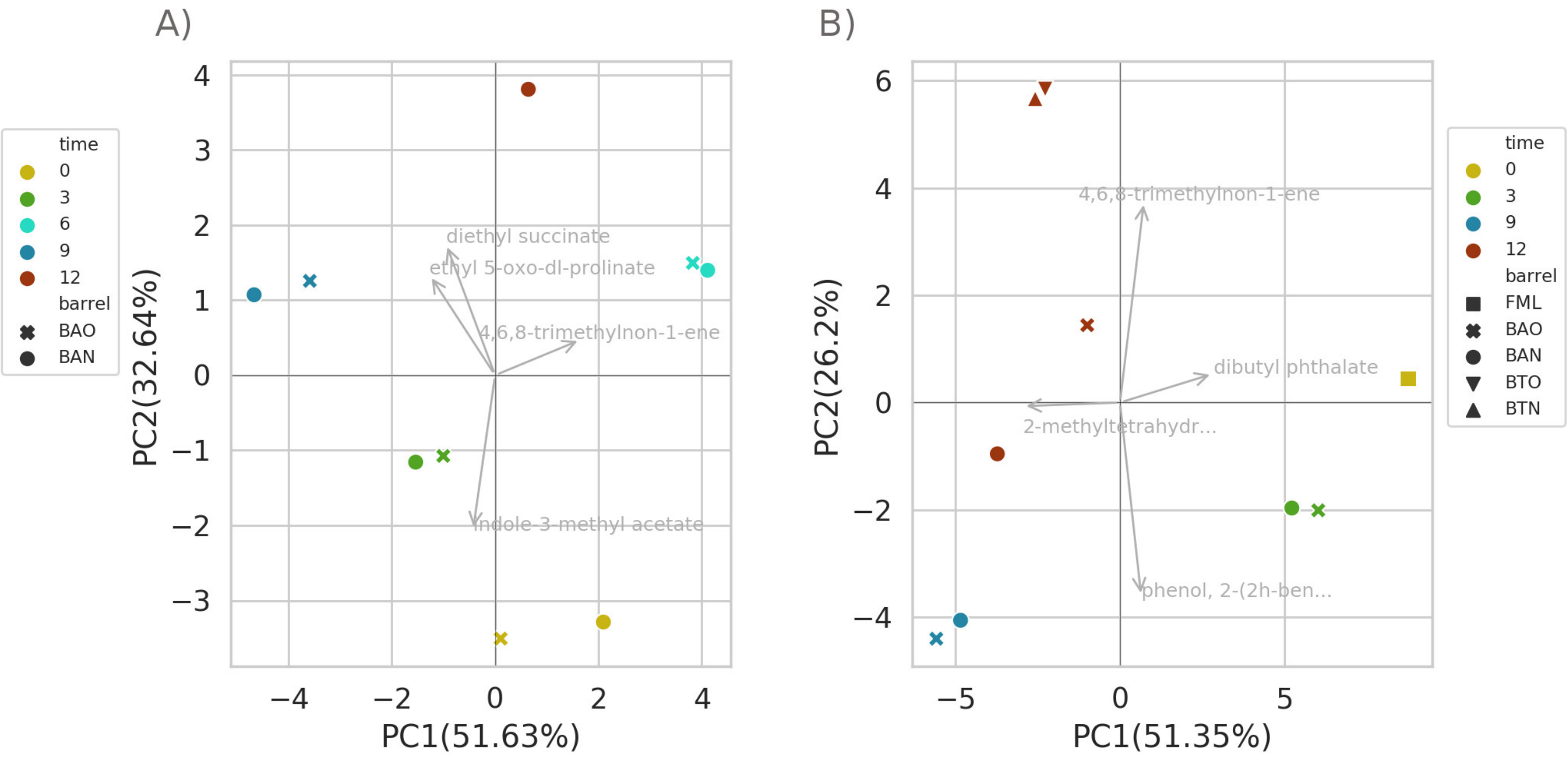

Figure S7: PCoA for cellars FB (A) and ICVV (B) based on the GC-MS metabolites. Metabolites with highest loadings acrosss the principal components are shown. Acronyms BAN and BAO refer to new and old barrel respectively, FML to final malolactic fermentation stage and BTN and BTO to bottle-aged wine from new and old barrel respectively.

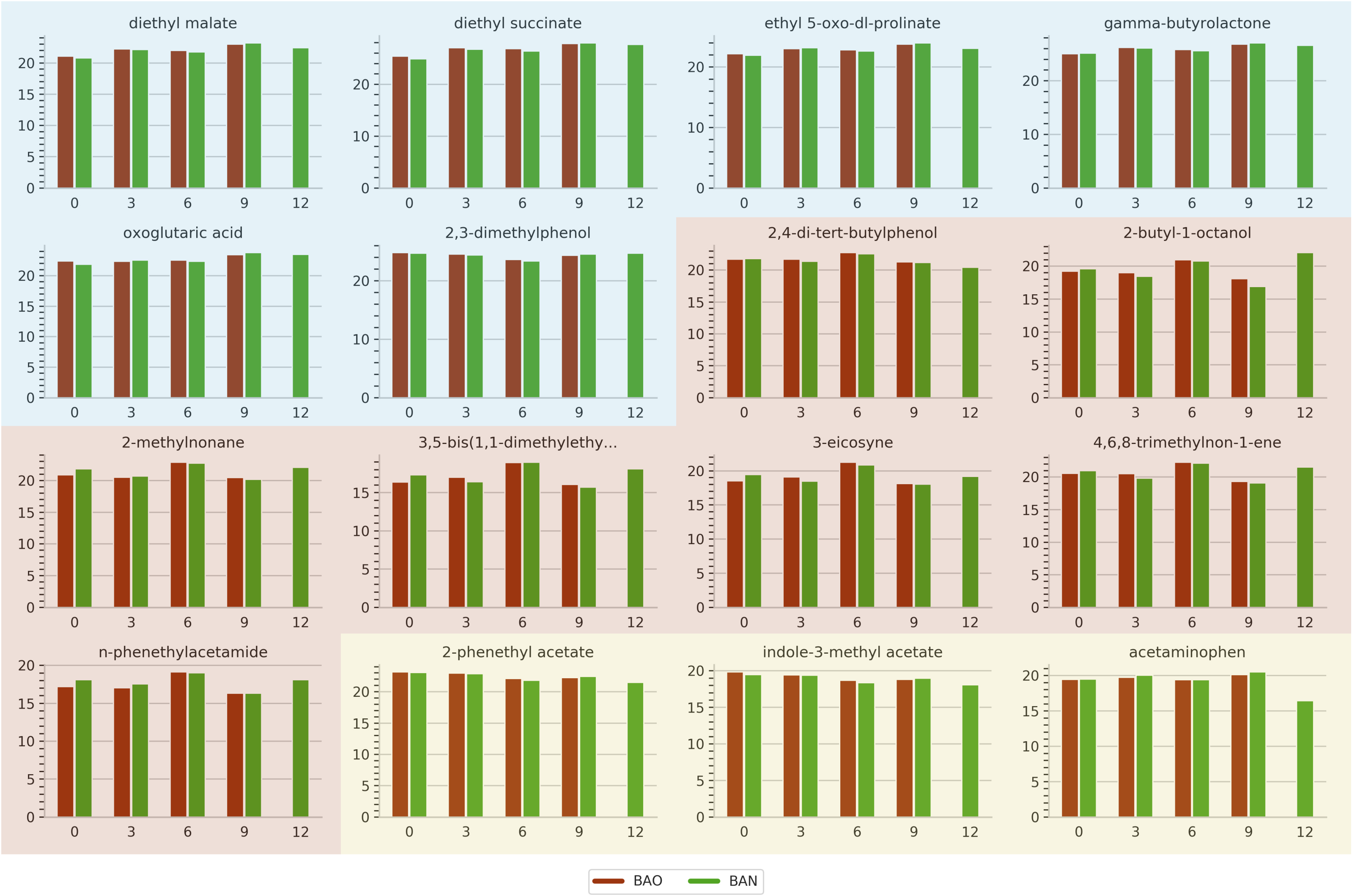

Figure S8: GC-MS log2 values of metabolites for cellar FB. Acronyms BAO and BAN refer to barrel-aged wine from old and new barrel respectively. Barplots of metabolites with blue background represent the group of metabolites whose concentration has an increasing trend, in orange background the group of metabolites with decreasing tendency and in red background the group of metabolites showing differences during the 6th and 12th month.

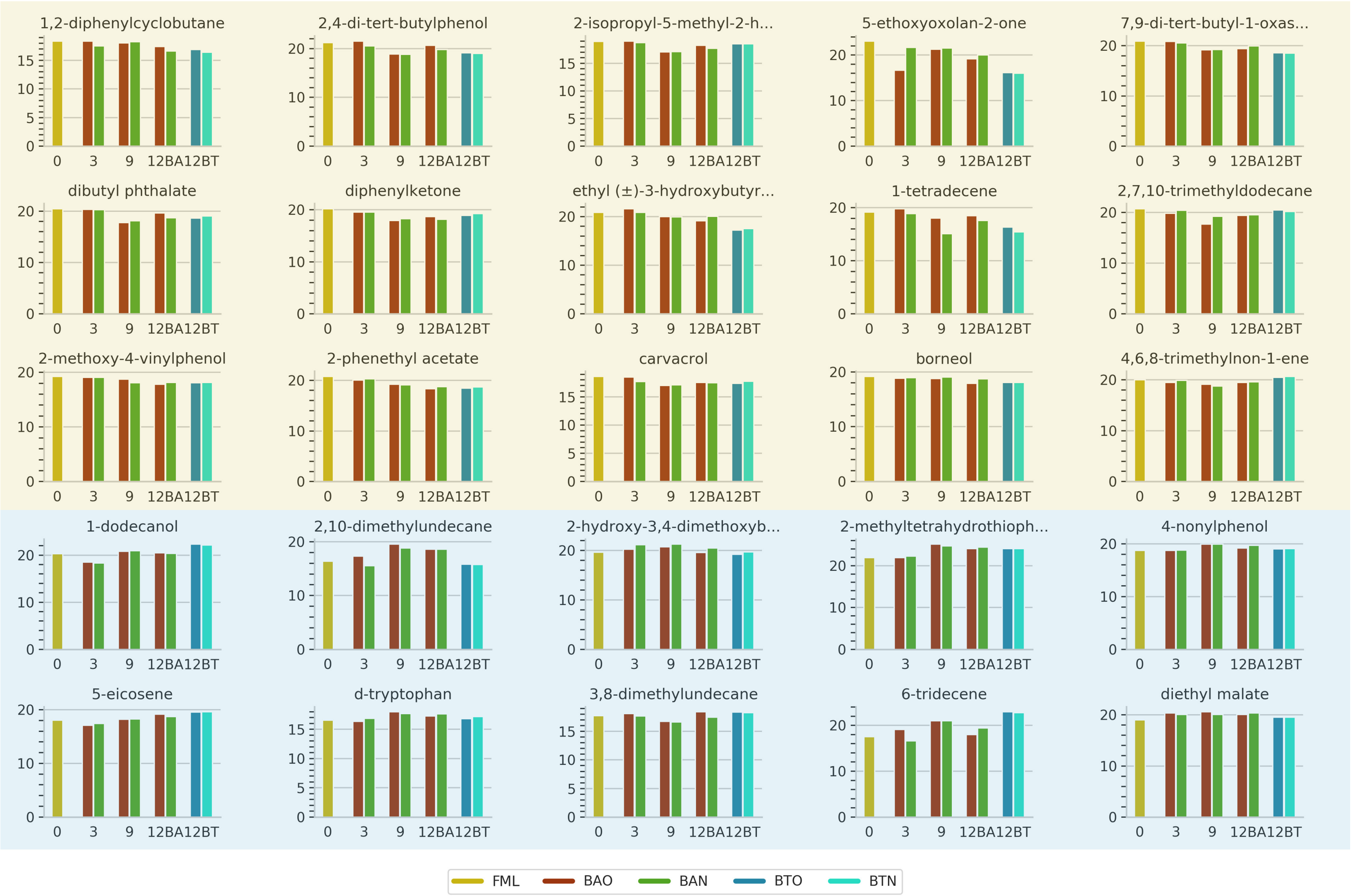

Figure S9: GC-MS log2 values of first 25 out of 48 metabolites for cellar ICVV. Acronyms BAN and BAO refer to new and old barrel respectively, FML to final malolactic fermentation stage and BTN and BTO to bottle-aged wine from new and old barrel respectively. Barplots of metabolites with blue background represent the group of metabolites whose concentration has an increasing trend whereas in orange background the group of metabolites with decreasing tendency.

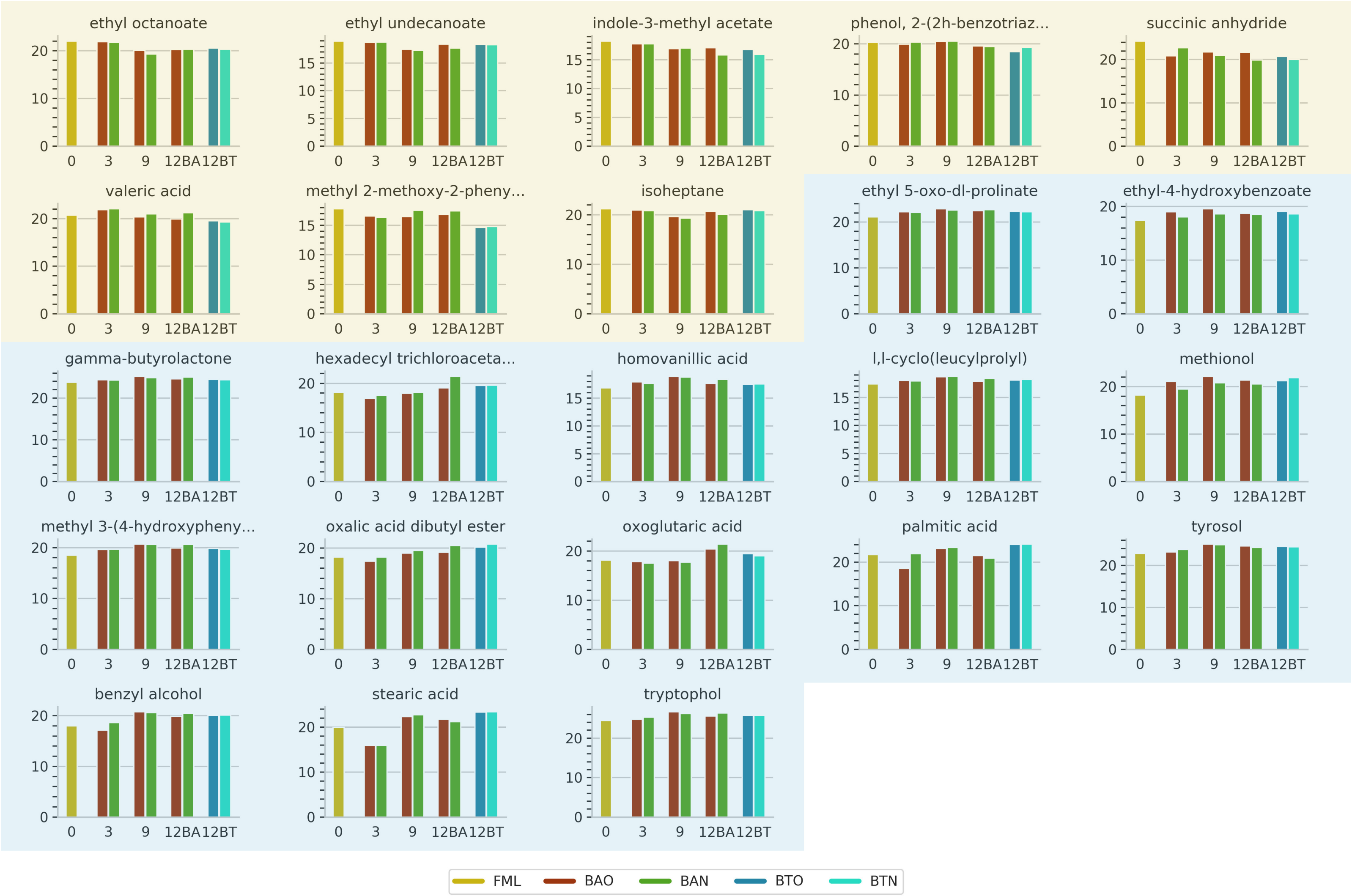

Figure S10: GC-MS log2 values of last 23 out of 48 metabolites for cellar ICVV. Acronyms BAN and BAO refer to new and old barrel respectively. Barplots of metabolites with blue background represent the group of metabolites whose concentration has an increasing trend whereas in orange background the group of metabolites with decreasing tendency.

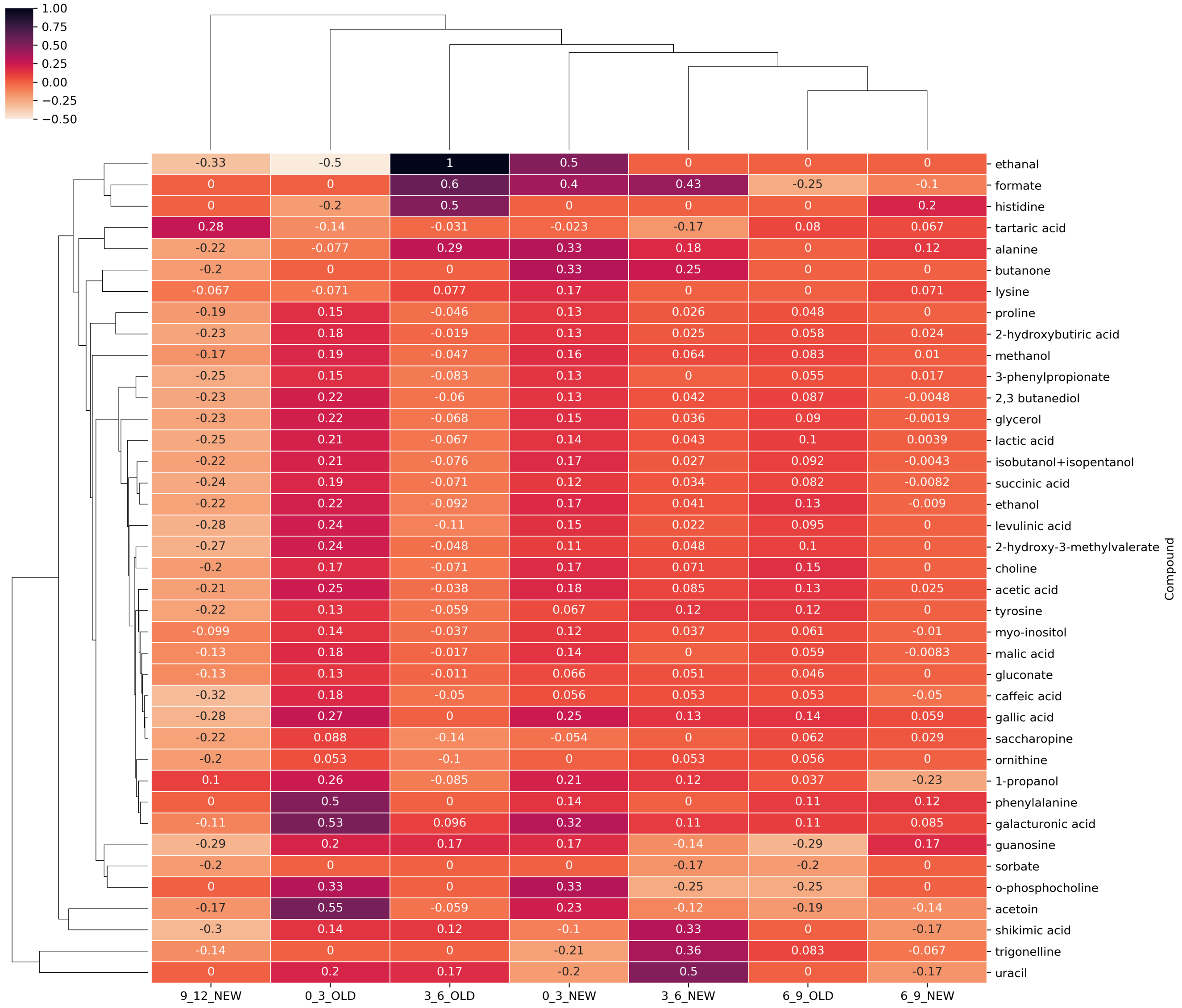

Figure S11: NMR growth rates heatmap for periods 0-3, 3-6, 6-9, and 9-12 based on barrel-type (OLD or NEW) for cellar FB. Values represent percentage of increase or decrease for each metabolite for the given period.

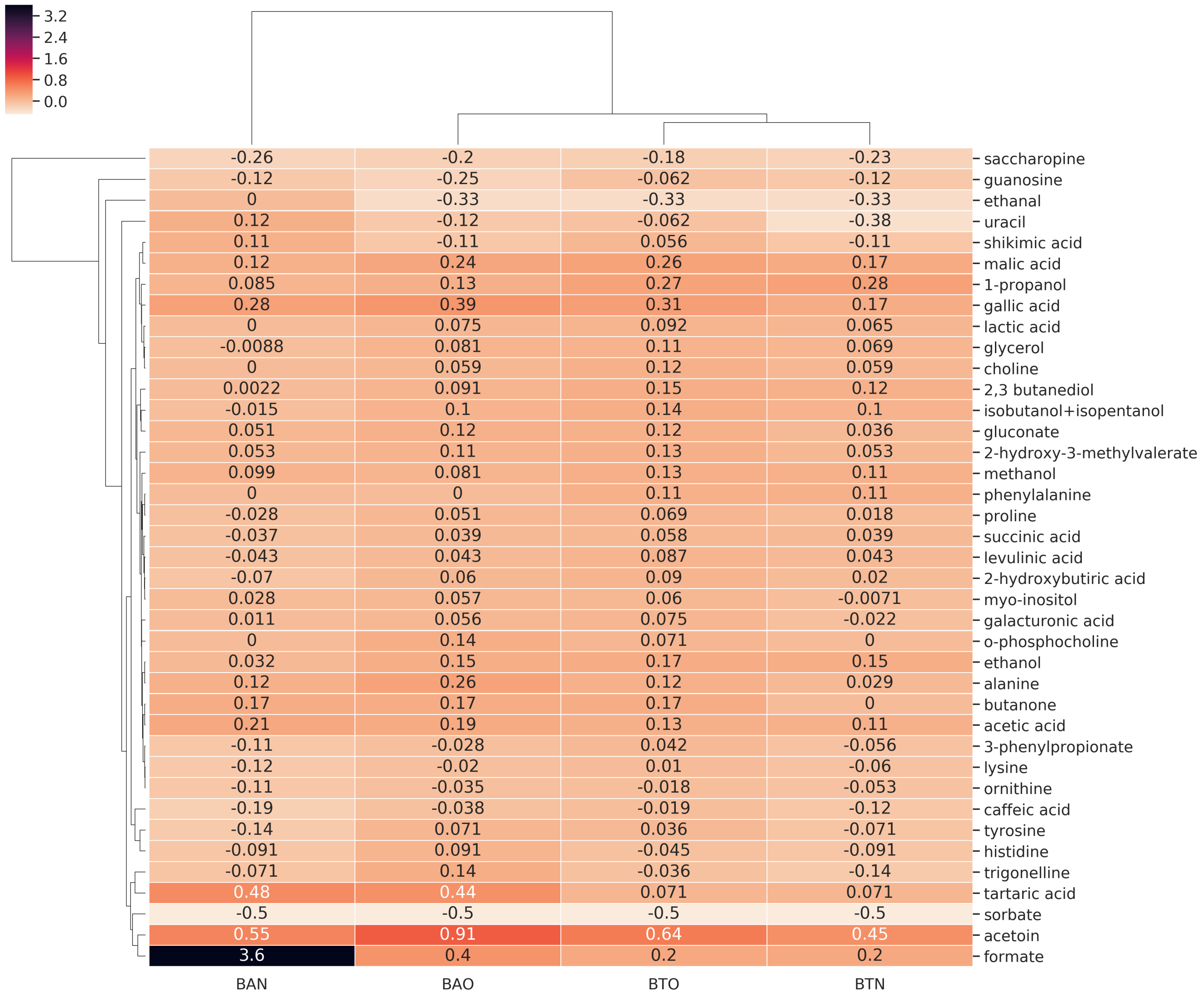

Figure S12: NMR growth rates for period 0-12 of barrel-aged wine from new (BAN) and old (BAO) barrel as well as of bottle-aged wine from new (BTN) and old (BTO) barrel for cellar ICVV. Values represent percentage of increase or decrease for each metabolite for the given period.

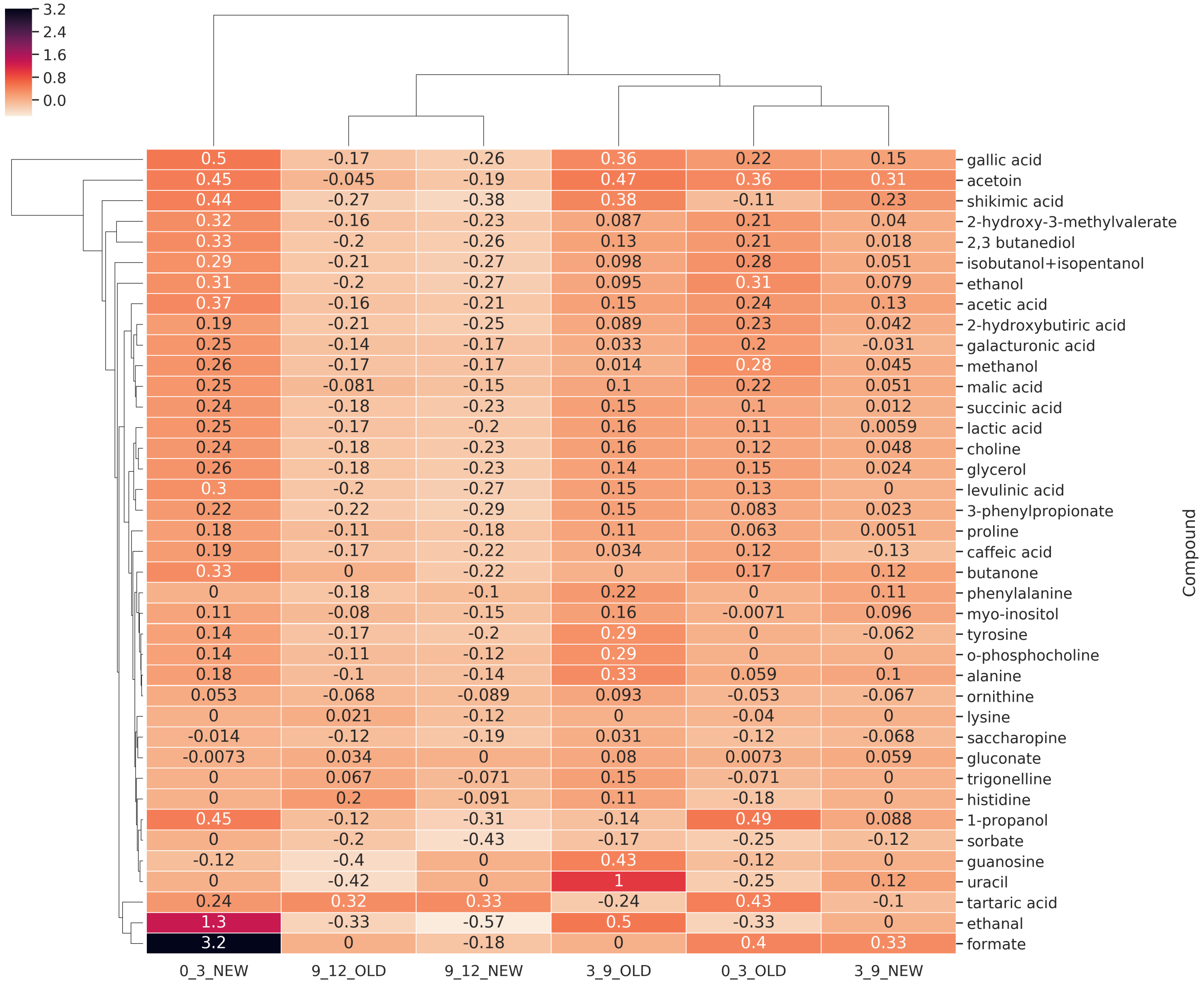

Figure S13: NMR growth rates for period 0-3, 3-9 and 9-12 based on barrel-type (OLD or NEW) for cellar ICVV. Values represent percentage of increase or decrease for each metabolite for the given period.

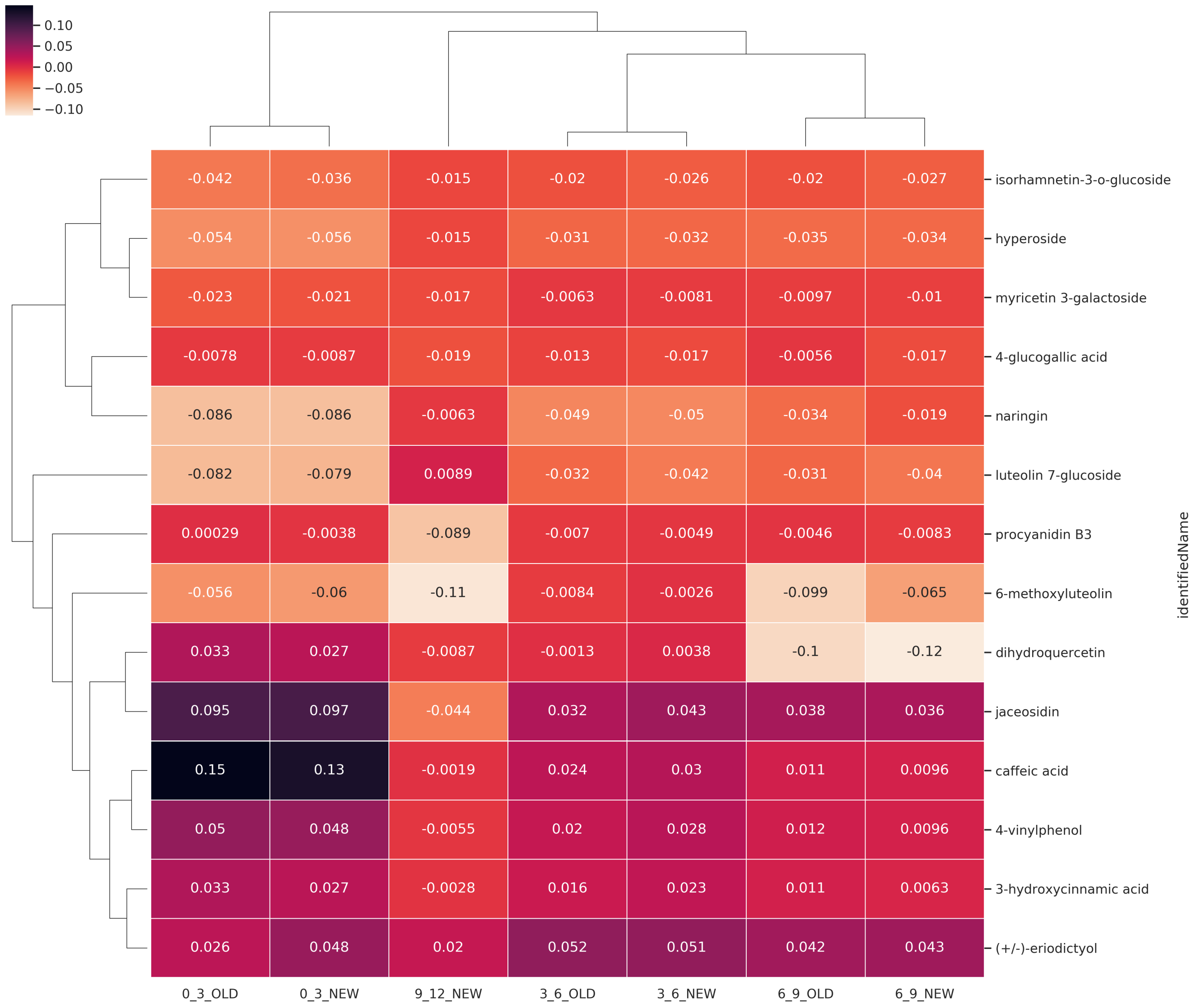

Figure S14: LC-MS growth rates heatmap for periods 0-3, 3-6, 6-9, and 9-12 based on barrel-type (OLD or NEW) for cellar FB. Values represent percentage of increase or decrease for each metabolite for the given period.

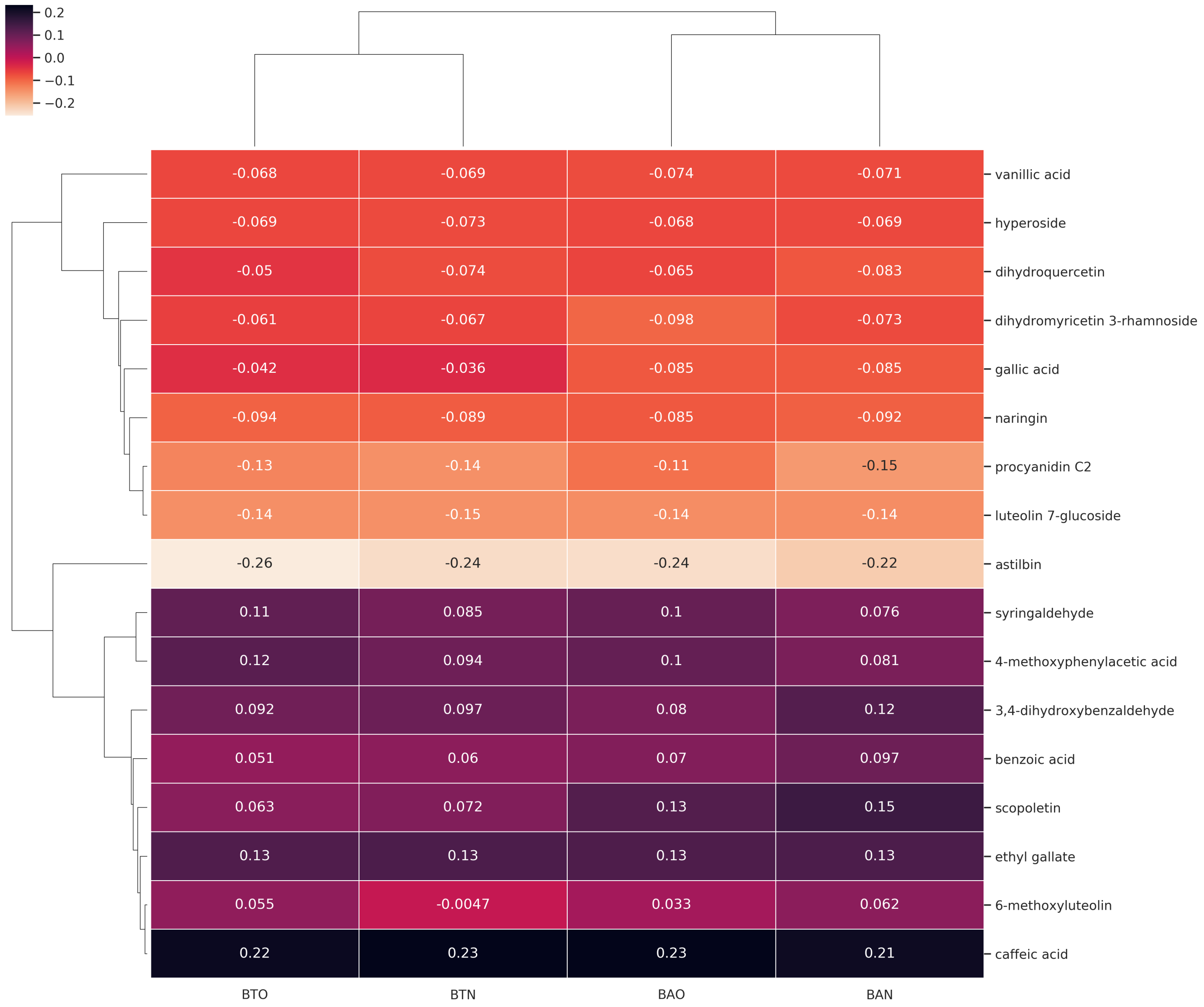

Figure S15: LC-MS growth rates for period 0-12 of barrel-aged wine from new (BAN) and old (BAO) barrel as well as of bottle-aged wine from new (BTN) and old (BTO) barrel for cellar ICVV. Values represent percentage of increase or decrease for each metabolite for the given period.

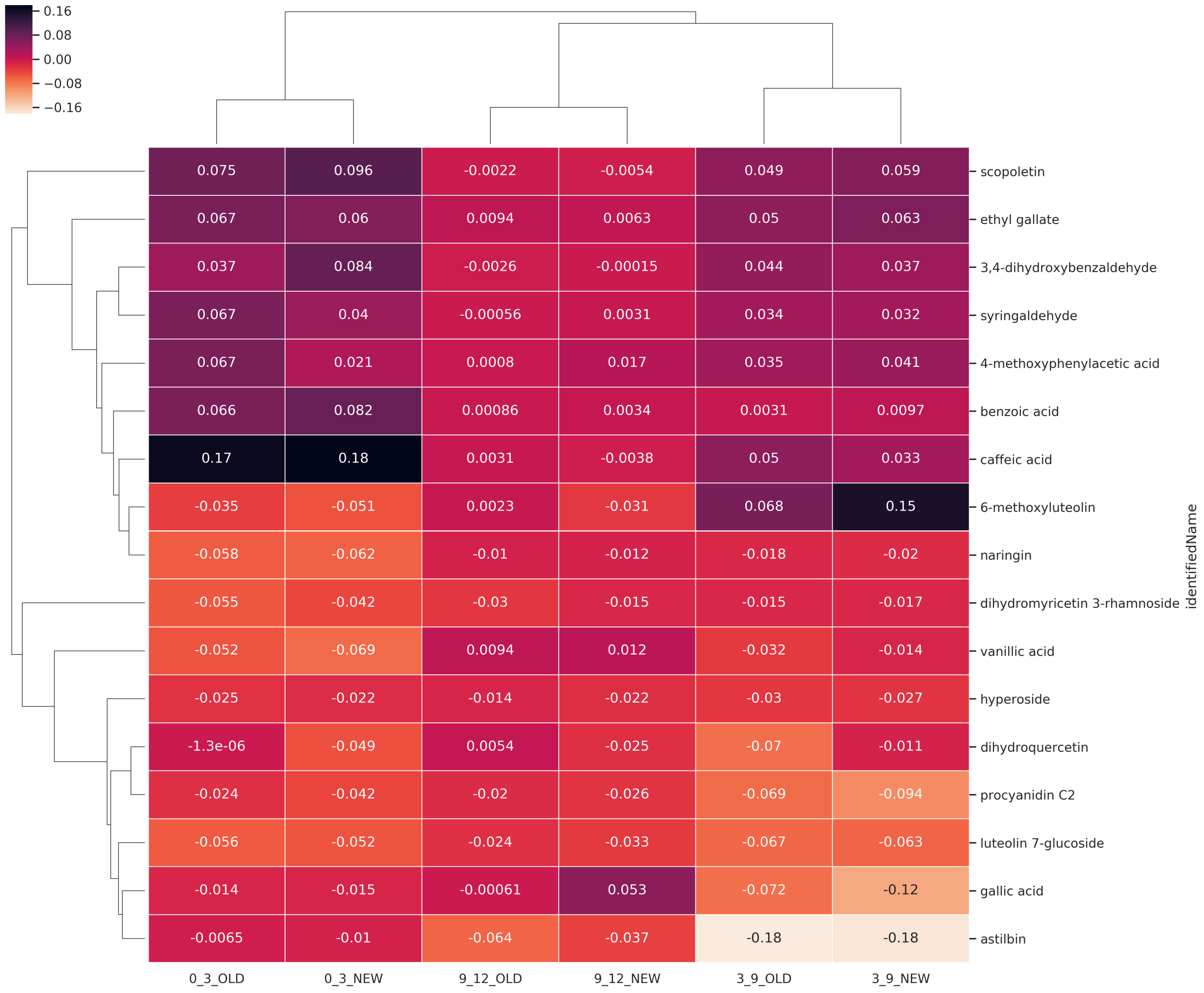

Figure S16: LC-MS growth rates for period 0-3, 3-9 and 9-12 based on barrel-type (OLD or NEW) for cellar ICVV. Values represent percentage of increase or decrease for each metabolite for the given period.

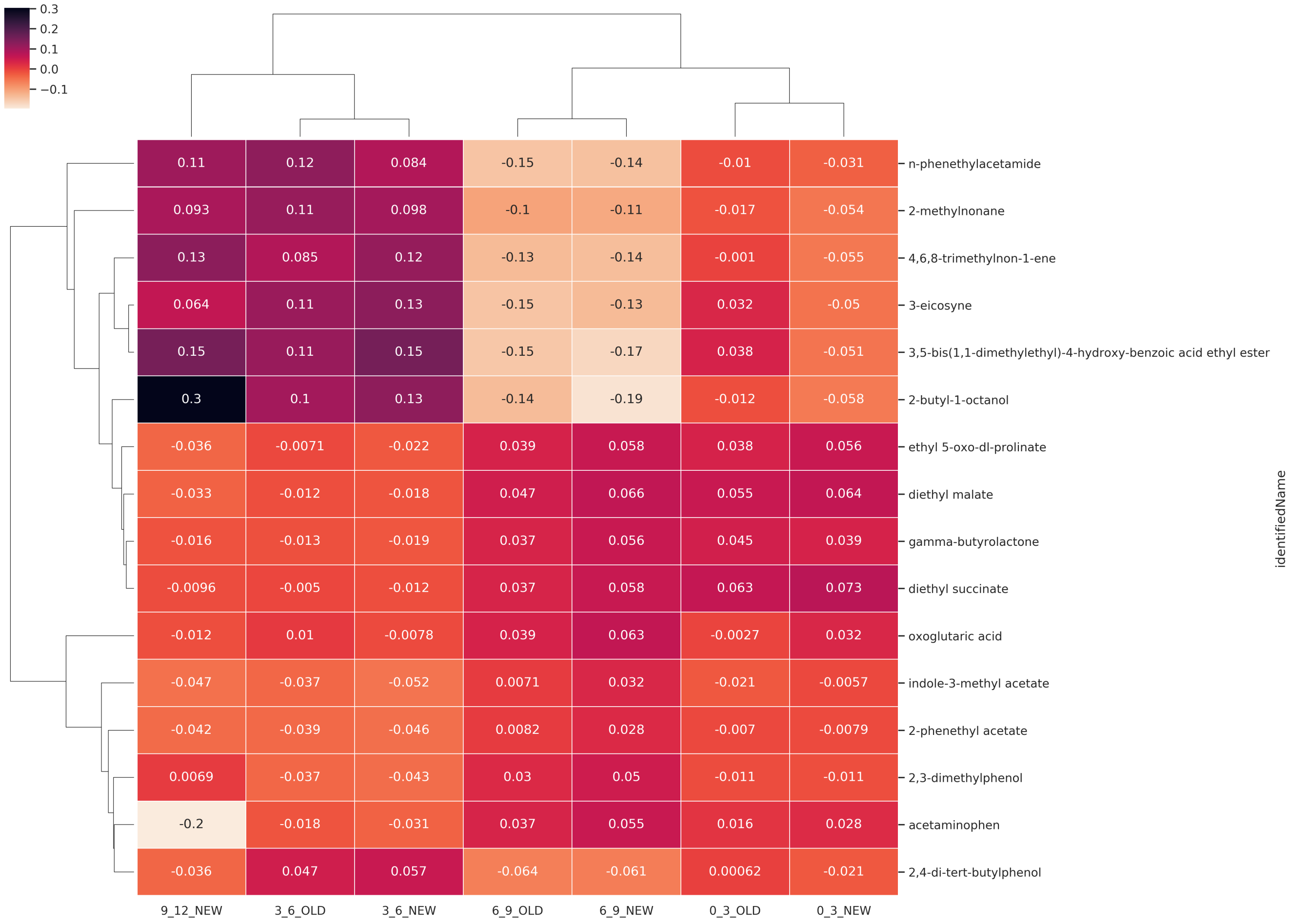

identifiedName	9_12_NEW	3_6_OLD	3_6_NEW	6_9_OLD	6_9_NEW	0_3_OLD	0_3_NEW
n-phenethylacetamide	0.11	0.12	0.084	-0.15	-0.14	-0.01	-0.031
2-methylnonane	0.093	0.11	0.098	-0.1	-0.11	-0.017	-0.054
4,6,8-trimethylnon-1-ene	0.13	0.085	0.12	-0.13	-0.14	-0.001	-0.055
3-eicosyne	0.064	0.11	0.13	-0.15	-0.13	0.032	-0.05
3,5-bis(1,1-dimethylethyl)-4-hydroxy-benzoic acid ethyl ester	0.15	0.11	0.15	-0.15	-0.17	0.038	-0.051
2-butyl-1-octanol	0.3	0.1	0.13	-0.14	-0.19	-0.012	-0.058
ethyl 5-oxo-dl-prolinate	-0.036	-0.0071	-0.022	0.039	0.058	0.038	0.056
diethyl malate	-0.033	-0.012	-0.018	0.047	0.066	0.055	0.064
gamma-butyrolactone	-0.016	-0.013	-0.019	0.037	0.056	0.045	0.039
diethyl succinate	-0.0096	-0.005	-0.012	0.037	0.058	0.063	0.073
oxoglutaric acid	-0.012	0.01	-0.0078	0.039	0.063	-0.0027	0.032
indole-3-methyl acetate	-0.047	-0.037	-0.052	0.0071	0.032	-0.021	-0.0057
2-phenethyl acetate	-0.042	-0.039	-0.046	0.0082	0.028	-0.007	-0.0079
2,3-dimethylphenol	0.0069	-0.037	-0.043	0.03	0.05	-0.011	-0.011
acetaminophen	-0.2	-0.018	-0.031	0.037	0.055	0.016	0.028
2,4-di-tert-butylphenol	-0.036	0.047	0.057	-0.064	-0.061	0.00062	-0.021

Figure S17: GC-MS growth rates heatmap for periods 0-3, 3-6, 6-9, and 9-12 based on barrel-type (OLD or NEW) for cellar FB. Values represent percentage of increase or decrease for each metabolite for the given period.

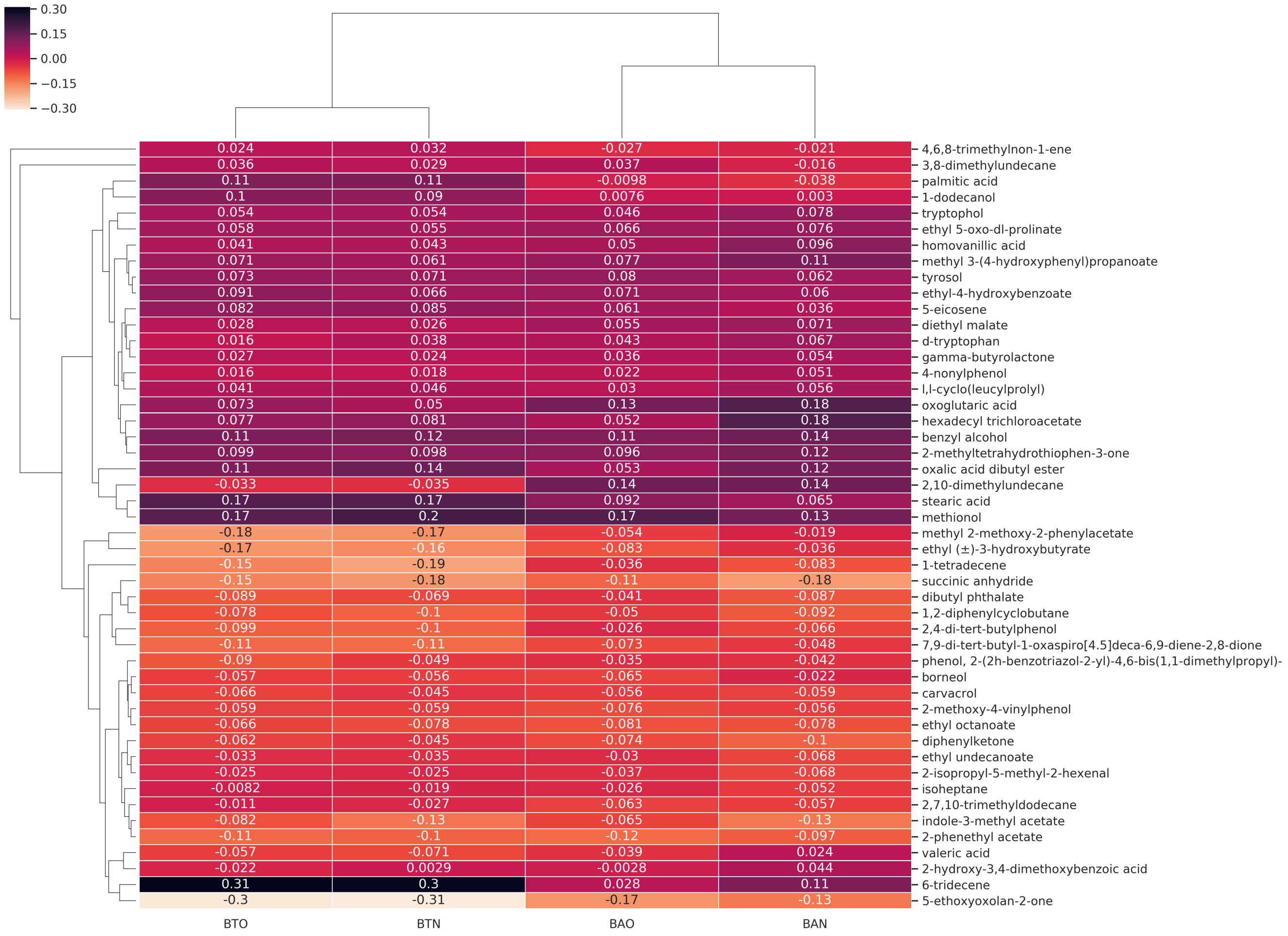

Figure S18: GC-MS growth rates for period 0-12 of barrel-aged wine from new (BAN) and old (BAO) barrel as well as of bottle-aged wine from new (BTN) and old (BTO) barrel for cellar ICVV. Values represent percentage of increase or decrease for each metabolite for the given period.

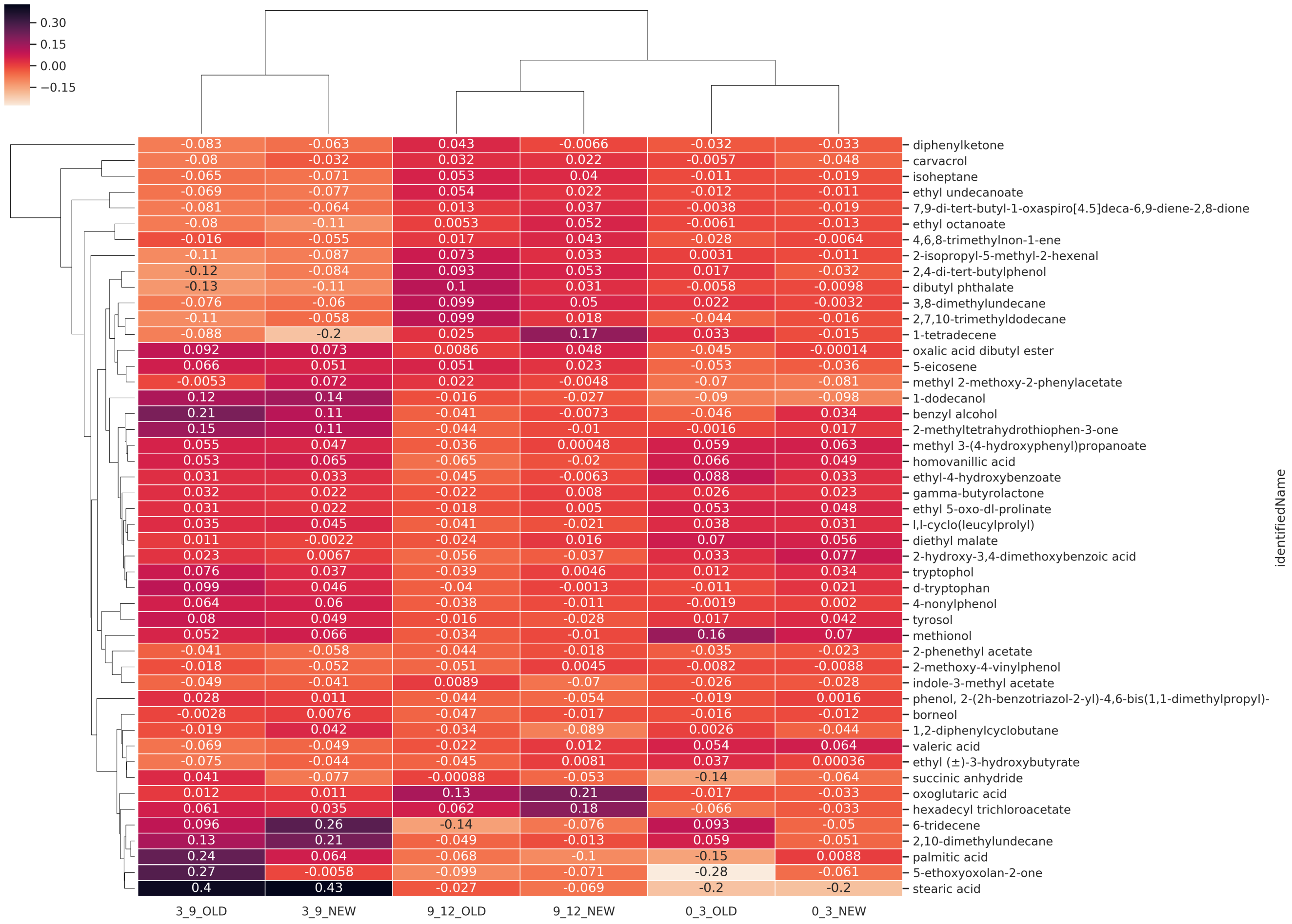

Figure S19: GC-MS growth rates for period 0-3, 3-9 and 9-12 based on barrel-type (OLD or NEW) for cellar ICVV. Values represent percentage of increase or decrease for each metabolite for the given period.
