# Supplementary material for: Qualitative Factor-Based Comparison of NMR, Targeted and Untargeted GC-MS and LC-MS on the Metabolomic Profiles of Rioja and Priorat Red Wines
Source: Foods. 2020 Sep 29;9(10):1381. doi: 10.3390/foods9101381 (PMC7600871; doi:10.3390/foods9101381)
Supplement: Supplementary file 1 [file foods-09-01381-s001.zip › supplementary_figures_tables 2/TableS3.pdf]

Table 2: LC-MS metabolites, with statistical and practical significance, for cellar FB. Numbers at the end of the acronyms represent sampling month. Values represent the area of the detected chromatographic peaks after applying variance stabilization. The metabolites can be grouped in the following chemical classes (1) benzene and substituted derivatives, (2) cinnamic acids and derivatives, (3) flavonoids and (4) organooxygen compounds. Metabolites that exhibited increasing tendency are indicated as (U) and those with decreasing tendency as (D).

Compound	BAO_0	BAO_3	BAO_6	BAO_9	BAN_0	BAN_3	BAN_6	BAN_9	BAN_12
4-vinylphenol[1U]	2,40E+05	4,45E+05	5,75E+05	6,79E+05	1,98E+05	3,54E+05	5,06E+05	5,74E+05	5,34E+05
3-hydroxycinnamic acid[2U]	4,68E+05	7,22E+05	8,99E+05	1,04E+06	4,25E+05	6,03E+05	8,16E+05	8,89E+05	8,55E+05
caffeic acid[2U]	9,37E+04	5,06E+05	6,97E+05	8,14E+05	9,42E+04	4,16E+05	6,11E+05	6,95E+05	6,77E+05
(+/-)-eriodictyol[3U]	5,96E+04	7,90E+04	1,42E+05	2,33E+05	4,88E+04	8,15E+04	1,45E+05	2,42E+05	3,09E+05
6-methoxyluteolin[3D]	2,99E+05	1,48E+05	1,34E+05	4,17E+04	2,79E+05	1,31E+05	1,27E+05	5,90E+04	1,72E+04
dihydroquercetin[3D]	3,19E+05	4,84E+05	4,76E+05	1,24E+05	3,05E+05	4,28E+05	4,50E+05	9,96E+04	9,02E+04
hyperoside[3D]	5,99E+06	2,58E+06	1,62E+06	9,89E+05	5,78E+06	2,42E+06	1,51E+06	9,39E+05	7,66E+05
isorhamnetin-3-o-glucoside[3D]	8,40E+05	4,77E+05	3,68E+05	2,86E+05	8,27E+05	5,06E+05	3,61E+05	2,57E+05	2,13E+05
jaceosidin[3U]	4,67E+04	1,30E+05	1,90E+05	3,03E+05	3,98E+04	1,12E+05	1,84E+05	2,86E+05	1,64E+05
luteolin 7-glucoside[3D]	3,37E+05	1,19E+05	8,16E+04	5,77E+04	3,23E+05	1,19E+05	7,26E+04	4,63E+04	5,09E+04
myricetin 3-galactoside[3D]	1,13E+07	7,72E+06	6,98E+06	5,99E+06	1,09E+07	7,70E+06	6,77E+06	5,74E+06	4,43E+06
naringin[3D]	1,82E+06	5,27E+05	2,76E+05	1,79E+05	1,79E+06	5,22E+05	2,69E+05	2,13E+05	1,97E+05
procyanidin B3[3D]	2,86E+05	2,87E+05	2,63E+05	2,48E+05	2,88E+05	2,75E+05	2,58E+05	2,33E+05	7,76E+04
4-glucogallic acid[4D]	2,25E+05	2,04E+05	1,75E+05	1,63E+05	2,35E+05	2,11E+05	1,71E+05	1,39E+05	1,11E+05
