# Supplementary material for: Qualitative Factor-Based Comparison of NMR, Targeted and Untargeted GC-MS and LC-MS on the Metabolomic Profiles of Rioja and Priorat Red Wines
Source: Foods. 2020 Sep 29;9(10):1381. doi: 10.3390/foods9101381 (PMC7600871; doi:10.3390/foods9101381)
Supplement: Supplementary file 1 [file foods-09-01381-s001.zip › supplementary_figures_tables 2/TableS2.pdf]

Table 1: NMR and targeted GC-MS metabolites for cellars FB and ICVV. Numbers at the end of the acronyms represent sampling month. NMR values correspond to concentrations which are expressed in arbitrary relative units, but in a perfect experimental could be taken as absolute molar concentration into the wine in mmols in comparison with the ERETIC signal of 0.6616 mM inserted in each spectra. Targeted GC-MS values are indicated with an asterisk and correspond to concentrations expressed in μg/L.

	FB									ICVV								
**Compound**	**BAO_0**	**BAO_3**	**BAO_6**	**BAO_9**	**BAN_0**	**BAN_3**	**BAN_6**	**BAN_9**	**BAN_12**	**FML_0**	**BAO_3**	**BAO_9**	**BAO_12**	**BAN_3**	**BAN_9**	**BAN_12**	**BTO_12**	**BTN_12**
ethanol	1153,66	1403,47	1274,93	1446,27	1203,49	1413,4	1470,88	1457,71	1137,7	1034,4	1357,82	1486,88	1184,62	1352,39	1459,15	1067,11	1212,08	1186,6
glycerol	67,8	82,85	77,2	84,14	71,46	82,48	85,42	85,26	65,65	68,31	78,66	89,96	73,81	86,26	88,32	67,71	75,575	73,03
1-propanol	0,47	0,59	0,54	0,56	0,47	0,57	0,64	0,49	0,54	0,47	0,7	0,6	0,53	0,68	0,74	0,51	0,595	0,6
isobutanol+isopentanol	1,85	2,23	2,06	2,25	1,91	2,24	2,3	2,29	1,79	1,36	1,74	1,91	1,5	1,75	1,84	1,34	1,555	1,5
2,3 butanediol	4,89	5,97	5,61	6,1	5,26	5,96	6,21	6,18	4,75	4,49	5,44	6,15	4,9	5,96	6,07	4,5	5,15	5,02
methanol	2,35	2,79	2,66	2,88	2,43	2,82	3	3,03	2,51	3,34	4,29	4,35	3,61	4,22	4,41	3,67	3,775	3,72
lactic acid	4,05	4,92	4,59	5,07	4,26	4,87	5,08	5,1	3,84	12,28	13,66	15,89	13,2	15,32	15,41	12,28	13,405	13,08
acetic acid	4,9	6,12	5,89	6,68	5,35	6,33	6,87	7,04	5,55	5,74	7,11	8,16	6,82	7,84	8,83	6,97	6,485	6,39
succinic acid	4,95	5,88	5,46	5,91	5,24	5,87	6,07	6,02	4,58	4,62	5,1	5,88	4,8	5,71	5,78	4,45	4,89	4,8
acetoin	0,11	0,17	0,16	0,13	0,13	0,16	0,14	0,12	0,1	0,11	0,15	0,22	0,21	0,16	0,21	0,17	0,18	0,16
2-hydroxybutiric acid	1,34	1,58	1,55	1,64	1,41	1,6	1,64	1,68	1,3	1	1,23	1,34	1,06	1,19	1,24	0,93	1,09	1,02
proline	1,32	1,52	1,45	1,52	1,34	1,51	1,55	1,55	1,26	4,95	5,26	5,86	5,2	5,83	5,86	4,81	5,29	5,04
butanone	0,04	0,04	0,04	0,04	0,03	0,04	0,05	0,05	0,04	0,06	0,07	0,07	0,07	0,08	0,09	0,07	0,07	0,06
tartaric acid	1,5	1,29	1,25	1,35	1,29	1,26	1,04	1,11	1,42	2,1	3,01	2,29	3,03	2,6	2,33	3,11	2,25	2,25
galacturonic acid	0,34	0,52	0,57	0,63	0,4	0,53	0,59	0,64	0,57	1,79	2,14	2,21	1,89	2,24	2,17	1,81	1,925	1,75
levulinic acid	0,38	0,47	0,42	0,46	0,4	0,46	0,47	0,47	0,34	0,23	0,26	0,3	0,24	0,3	0,3	0,22	0,25	0,24
malic acid	1,02	1,2	1,18	1,25	1,06	1,21	1,21	1,2	1,04	1,1	1,34	1,48	1,36	1,37	1,44	1,23	1,385	1,29
lysine	0,14	0,13	0,14	0,14	0,12	0,14	0,14	0,15	0,14	0,5	0,48	0,48	0,49	0,5	0,5	0,44	0,505	0,47
sorbate	0,05	0,05	0,05	0,04	0,06	0,06	0,05	0,05	0,04	0,08	0,06	0,05	0,04	0,08	0,07	0,04	0,04	0,04
2-hydroxy-3-methylvalerate	0,17	0,21	0,2	0,22	0,19	0,21	0,22	0,22	0,16	0,19	0,23	0,25	0,21	0,25	0,26	0,2	0,215	0,2
ornithine	0,19	0,2	0,18	0,19	0,19	0,19	0,2	0,2	0,16	0,57	0,54	0,59	0,55	0,6	0,56	0,51	0,56	0,54
choline	0,12	0,14	0,13	0,15	0,12	0,14	0,15	0,15	0,12	0,17	0,19	0,22	0,18	0,21	0,22	0,17	0,19	0,18
o-phosphocholine	0,03	0,04	0,04	0,03	0,03	0,04	0,03	0,03	0,03	0,07	0,07	0,09	0,08	0,08	0,08	0,07	0,075	0,07
trigonelline	0,12	0,12	0,12	0,13	0,14	0,11	0,15	0,14	0,12	0,14	0,13	0,15	0,16	0,14	0,14	0,13	0,135	0,12
histidine	0,05	0,04	0,06	0,06	0,05	0,05	0,05	0,06	0,06	0,11	0,09	0,1	0,12	0,11	0,11	0,1	0,105	0,1
myo-inositol	1,65	1,88	1,81	1,92	1,67	1,87	1,94	1,92	1,73	1,41	1,4	1,62	1,49	1,56	1,71	1,45	1,495	1,4
tyrosine	0,15	0,17	0,16	0,18	0,15	0,16	0,18	0,18	0,14	0,14	0,14	0,18	0,15	0,16	0,15	0,12	0,145	0,13
gallic acid	0,11	0,14	0,14	0,16	0,12	0,15	0,17	0,18	0,13	0,18	0,22	0,3	0,25	0,27	0,31	0,23	0,235	0,21
shikimic acid	0,07	0,08	0,09	0,09	0,1	0,09	0,12	0,1	0,07	0,09	0,08	0,11	0,08	0,13	0,16	0,1	0,095	0,08
caffeic acid	0,17	0,2	0,19	0,2	0,18	0,19	0,2	0,19	0,13	0,26	0,29	0,3	0,25	0,31	0,27	0,21	0,255	0,23
formate	0,05	0,05	0,08	0,06	0,05	0,07	0,1	0,09	0,09	0,05	0,07	0,07	0,07	0,21	0,28	0,23	0,06	0,06
phenylalanine	0,06	0,09	0,09	0,1	0,07	0,08	0,08	0,09	0,09	0,09	0,09	0,11	0,09	0,09	0,1	0,09	0,1	0,1

3-phenylpropionate	0,52	0,6	0,55	0,58	0,52	0,59	0,59	0,6	0,45	0,36	0,39	0,45	0,35	0,44	0,45	0,32	0,375	0,34
uracil	0,05	0,06	0,07	0,07	0,05	0,04	0,06	0,05	0,05	0,08	0,06	0,12	0,07	0,08	0,09	0,09	0,075	0,05
guanosine	0,05	0,06	0,07	0,05	0,06	0,07	0,06	0,07	0,05	0,08	0,07	0,1	0,06	0,07	0,07	0,07	0,075	0,07
gluconate	1,57	1,77	1,75	1,83	1,67	1,78	1,87	1,87	1,62	1,37	1,38	1,49	1,54	1,36	1,44	1,44	1,53	1,42
alanine	0,26	0,24	0,31	0,31	0,21	0,28	0,33	0,37	0,29	0,34	0,36	0,48	0,43	0,4	0,44	0,38	0,38	0,35
saccharopine	0,34	0,37	0,32	0,34	0,37	0,35	0,35	0,36	0,28	0,74	0,65	0,67	0,59	0,73	0,68	0,55	0,605	0,57
ethanal	0,02	0,01	0,02	0,02	0,02	0,03	0,03	0,03	0,02	0,03	0,02	0,03	0,02	0,07	0,07	0,03	0,02	0,02
4-ethylphenol *	28	38	31	98	2	2	17	66	46	5	11	11	11	2	2	23	3,5	3
4-ethylguaiacol *	0	1	2	6	0	1	3	7	4	0	0	0	0	0	2	0	0	0
5-methylfurfural*	0	7	9	6	36	106	63	58	55	0	7	5	5	675	1134	358	0	91
