# Supplementary material for: Qualitative Factor-Based Comparison of NMR, Targeted and Untargeted GC-MS and LC-MS on the Metabolomic Profiles of Rioja and Priorat Red Wines
Source: Foods. 2020 Sep 29;9(10):1381. doi: 10.3390/foods9101381 (PMC7600871; doi:10.3390/foods9101381)
Supplement: Supplementary file 1 [file foods-09-01381-s001.zip › supplementary_figures_tables 2/Supplementary table 1.pdf]

Supplementary table 1. Quality parameters for GC targeted analysis.

Compound	r^2	LoD (µg/L)	LoQ (µg/L)	Recovery (%)	Accuracy (%)	Repeatability (%RSD, *n*=5)
**4-EP**	0.9960	0.3	1.0	116	109	2.2
**4-EG**	0.9952	0.4	1.2	103	108	2.2
**5-MF**	0.9975	0.8	2.6	111	101	3.9
**2-MIB**	0.9997	2.6	8.7	110	99	8.8
**5-HMF**	0.9933	3.3	11.1	66	101	5.5
